# Predictors of primary breast cancers responsiveness to preoperative Epirubicin/Cyclophosphamide-based chemotherapy: translation of microarray data into clinically useful predictive signatures

**DOI:** 10.1186/1479-5876-3-32

**Published:** 2005-08-09

**Authors:** Olga Modlich, Hans-Bernd Prisack, Marc Munnes, Werner Audretsch, Hans Bojar

**Affiliations:** 1 Institute of Chemical Oncology, University of Düsseldorf, Düsseldorf, Germany; 2 Bayer Healthcare AG, Diagnostic Research Germany, Leverkusen, Germany; 3 Interdisciplinary Breast Center IBC, City Hospital, Düsseldorf, Germany

**Keywords:** breast cancer, preoperative chemotherapy, microarray, prognostic classification

## Abstract

**Background:**

Our goal was to identify gene signatures predictive of response to preoperative systemic chemotherapy (PST) with epirubicin/cyclophosphamide (EC) in patients with primary breast cancer.

**Methods:**

Needle biopsies were obtained pre-treatment from 83 patients with breast cancer and mRNA was profiled on Affymetrix HG-U133A arrays. Response ranged from pathologically confirmed complete remission (pCR), to partial remission (PR), to stable or progressive disease, "No Change" (NC). A primary analysis was performed in breast tissue samples from 56 patients and 5 normal healthy individuals as a training cohort for predictive marker identification. Gene signatures identifying individuals most likely to respond completely to PST-EC were extracted by combining several statistical methods and filtering criteria. In order to optimize prediction of non responding tumors Student's *t*-test and Wilcoxon test were also applied. An independent cohort of 27 patients was used to challenge the predictive signatures. A *k*-Nearest neighbor algorithm as well as two independent linear partial least squares determinant analysis (PLS-DA) models based on the training cohort were selected for classification of the test samples. The average specificity of these predictions was greater than 74% for pCR, 100% for PR and greater than 62% for NC. All three classification models could identify all pCR cases.

**Results:**

The differential expression of 59 genes in the training and the test cohort demonstrated capability to predict response to PST-EC treatment. Based on the training cohort a classifier was constructed following a decision tree.

First, a transcriptional profile capable to distinguish cancerous from normal tissue was identified. Then, a "favorable outcome signature" (31 genes) and a "poor outcome signature" (26 genes) were extracted from the cancer specific signatures. This stepwise implementation could predict pCR and distinguish between NC and PR in a subsequent set of patients. Both PLS-DA models were implemented to discriminate all three response classes in one step.

**Conclusion:**

In this study signatures were identified capable to predict clinical outcome in an independent set of primary breast cancer patients undergoing PST-EC.

## Introduction

Breast cancer is the most common neoplasia of women being diagnosed in approximately 211,000 women annually in the United States. In spite of earlier detection and improved treatment, it remains the second leading cause of cancer-related death in the United States and in other developed countries [1]. The genetic background of patients and the tumor's genetic and epigenetic anomalies create, in combination, molecularly distinct subtypes arising from distinct cell types within the ductal epithelium [2,3]. This genetic complexity underlies the clinical heterogeneity of breast cancer limiting a rational selection of treatment tailored to individual patient/tumor characteristics.

Standard therapeutic decision-making (*i.e.*., NIH; St. Gallen consensus) rely on several clinicopathological factors such as patients' age, tumor stage, grade, size, nodal status as well as hormone, and growth receptor status. The analysis of single molecular markers such as ki-67 and ERBB2 can also contribute to the therapeutic decision making. Although all of these factors have been correlated to patients' survival in general, the same prognostic profile often results in dissimilar clinical outcomes in individual patients. Thus, conventional prognostic factors provide insufficient information to evaluate the heterogeneity of this disease and to make treatment more effective for individual patients.

One problem faced by present cancer therapy is the over-treatment of patients with chemotherapy, which is associated with severe toxicity and increasing healthcare spending without clear survival benefit over untreated controls [4,5]. Because of the lack of adequate predictive markers (*i.e.*, ER, HER2/neu), nearly all patients receive routinely standard treatment in spite of grim changes of deriving any benefit. Therefore, the identification of molecular markers predictive of patients' responsiveness to treatment is becoming a central focus of translational research.

Micro array technology offers insights about the simultaneous expression of thousands of genes providing global information about the transcriptional program associated with specific cellular or tissue conditions. This provides a high-throughput screening tool for the identification of molecular patterns of cancerous cell possibly associated with their sensitivity to therapy [6-9]. This strategy yielded significant contributions by dissecting beyond histopathologic features the molecular aspects of breast cancers, their association with lymph-nodal spread, metastatization and overall survival.

An important and so far seldom explored utilization of micro array technology is the identification of signatures predictive of responsiveness to chemotherapy. To get clear correlation between chemotherapeutic success and pre treatment gene expression we needed to rely in a model where chemotherapy is given before surgical resection so that its outcome could be evaluated. Beside the most common postoperative (adjuvant) chemotherapy, preoperative systemic therapy (PST) has been recently proposed for early-stage breast cancer. PST, uses cytotoxic drugs as the first modality of treatment allowing *in vivo *monitoring of the therapeutic responsiveness of a primary tumor over a given time period (*e.g*., 4 months). PST is offered preoperatively to patients with either large inoperable breast cancers or to patients interested in breast conserving surgery [10-16]. PST in general does not offer a survival advantage over standard adjuvant treatment but does identify those patients (up to 20%) with tumors reacting with a complete remission to the drug [17,18]. Complete tumor remission, as confirmed by pathological examination, is often associated with prolonged disease free survival [19-22]. Additionally, PST can reduce the growth rate of residual distant micrometastases compared with classical adjuvant therapy [17].

By predicting which subset of tumors may respond to PST transcriptional profiling of pre-treatment samples could represent a powerful tool for patient selection.

## Patients, Materials and methods

### Patients

This study was performed in collaboration with the Institute of Chemical Oncology, University of Düsseldorf, Germany, and Bayer Healthcare AG, Diagnostic Research, Leverkusen, Germany. All patients were recruited at the Interdisciplinary Breast Center IBC, City Hospital Düsseldorf. Patients signed an informed consent before any procedure. Study eligibility criteria required that participants presented with not previously treated primary breast cancer to be treated preoperatively.

Samples of primary breast carcinomas were collected between May 1999 and March 2003 from patients subjected to PST treatment with epirubicine/cyclophosphamide (EC). Since in several cases treatment modifications occurred or full pathological confirmation of response status was not conclusive not all samples were studied. Quality of samples related to delays in processing also limited the number of samples studied. In the end, a total of 56 tumor samples were identified from comparable treatment groups and were studied for marker discovery together with five normal breast samples excised from patient with benign pathology. Additionally, tumor samples removed from 27 patients treated with EC-based PST between December 2002 and September 2003 were analyzed as a second independent validation cohort. EC consisted of epirubicin 90 mg m^2 ^per day 1 in a short i.v. infusion, and cyclophosphamide 600 mg m^2 ^per day 1 in a short i.v. infusion. Four cycles of EC were administrated 14 days apart. Some patients received additionally Tamoxifen, Femara or seldom Zoladex for 4–5 weeks after EC course and before surgery. All tumor samples were collected as needle biopsies of primary tumors prior to any treatment. The biopsies were obtained under local anesthesia using Bard^® ^MAGNUM™ Biopsy Instrument (C. R. Bard, Inc., Covington, U. S.) with Bard^® ^Magnum biopsy needles (BIP GmbH, Tuerkenfeld, Germany) following ultrasound guidance. Samples were collected following routine conditions for pathological diagnosis following institutional review board guidelines. Pathological examination was carried out for all tumor samples by the same pathologist at the Interdisciplinary Breast Center IBC. The remainders of the samples were flash-frozen. After PST, all patients underwent a radical mastectomy or a lumpectomy and axillary node dissection at the discretion of the treating breast surgeon. Postoperative chemotherapy was administrated at the discretion of the treating medical oncologist. Breast or chest wall irradiation was administrated in selected patients. In addition, all women with ER-positive tumors were started on tamoxifen therapy. A detailed list of all samples and clinical data is presented in Tables 1 and 2 (see also Additional files 1 and 2). Additionally, five normal breast samples from reduction mammoplasties were analyzed.

**Table 1:** Clinical and molecular data on breast cancer patients (training set).

Case	Response	Tumor reduction,%	Histology pre	Age	ER	PR	BCL2	P53	KI67,%	CerbB2	Grading
BC1492	NC	0	invasive lobular	50	0	0	0	1	28	0	2
BC1426	NC	0	invasive ductal + intraductal(40%)	62	1	1	400	0	2	1+ to 2+	1 a. 2
BC1257	NC	0	invasive lobular	69	1	1	180	0	16	1+ to 2+	2
BC1176	NC	0	invasive lobular a. tubular-lobular	47	1	1	130	0	7	0	2
BC1092	NC	0	invasive lobular a. ductal	66	1	1	0	0	10	0	2
BC1050	NC	0	invasive tubular-lobular; multifocal	60	1	1	60	0	3	0	2
BC1034	NC	0	intraductal a. invasive	43	1	1	0	0	2	0	2
BC1044	NC	0	invasive tubular-lobular	68	1	1	70	0	6	0	1
BC1466	pCR	100	invasive ductal a. intraductal(30%)	57	0	0	0	0	26	3+	2
BC1255	pCR	100	invasive lobular	57	1	0	60	1	26	0	2
BC1254	pCR	100	medullary	62	0	0	0	0	70	1+	3
BC1180	pCR	100	invasive lobular a. ductal	32	0	0	0	0	20	1+	2
BC1159	pCR	100	invasive lobular a. ductal	40	0	0	70	0	70	1+ to 2+	2
BC1042	pCR	100	non-typical medullary a. intraductal (bifocal)	38	0	0	10	0	35	0	3
BC1032	pCR	100	invasive lobular a. ductal	58	0	0	0	0	15	3+	2
BC1443	PR/CR	94	invasive lobular a. ductal	61	1	0	10	0	30	3+	2
BC1167	PR	0	invasive lobular	71	1	1	400	0	8	0	2
BC1162	PR	0	invasive lobular	66	1	1	70	0	7	1+	2
BC1143	PR	0	invasive ductal a. intraductal(5%)	54	1	1	210	0	17	1+	2
BC1138	PR	0	invasive lobular; multifocal	57	1	1	80	0	13	1+	2
BC1100	PR	0	invasive tubular-lobular	55	1	1	140	0	4	0	1
BC1040	PR	0	invasive ductal	40	1	1	294	0	28	0	2
BC1170	PR	0	invasive lobular a. ductal bifocal	67	1	0	210	0	4	2+ to 3+	2
BC1140	PR	10	left: invasive lobular	73	1	1	180	0	14	0	2
BC1418	PR	12	left:bifocal invasive tubular-lobular	57	1	0	300	0	7	2+	1
BC1420	PR	15	invasive ductal a. lobular; multifocal	63	1	1	400	0	3	2+	2
BC1491	PR	18	invasive lobular	64	1	1	300	0	7	0	2
BC1515	PR	20	invasive ductal	64	1	1	300	1	10	0	2
BC1445	PR	20	right:invasive lobular; bifocal	64	1	0	160	0	15	1+ to 2+	2
BC1036	PR	24	invasive ductal	58	1	1	392	0	15	0	2
BC1308	PR	25	invasive lobular; multifocal	74	0	0	0	0	16	2+	2
BC1133	PR	25	invasive ductal	53	0	0	294	0	50	0	3
BC1259	PR	32	invasive ductal a. lobular (Herd1) a. invasive ductal (Herd2)	59	1	0	300	0	16	2+ to 3+	2
BC1498	PR	33	invasive ductal; bifocal	62	1	0	15	0	19	3+	3
BJ_40613	PR	35	invasive ductal	61	1	1	100	0	4	2+	2
BC1166	PR	35	invasive ductal a. lobular	45	1	1	140	0	14	1+ to 2+	2
BC1142	PR	35	invasive lobular; bifocal	53	1	1	20	0	18	1+	2
BC1422	PR	40	invasive ductal a. lobular; multifocal	53	1	1	360	0	2	2+	2
BC1132	PR	40	invasive ductal	41	1	1	30	1	18	2+	3
BC1096	PR	40	invasive ductal	46	1	1	294	0	13	0	2
BC1129	PR	42	invasive tubular-lobular	52	0	0	0	0	15	2+	2
BC1130	PR	45	invasive lobular	42	1	1	140	0	NA	0	2
BC1131	PR	45	invasive ductal (pulmonal, ossar)	63	1	0	300	0	45	0	2
BC1256	PR	50	invasive lobular	60	0	0	0	1	17	1+	2
BC1446	PR	50	invasive lobular; bifocal	53	1	1	140	0	35	1+	2
BC1116	PR	53	invasive lobular	49	1	1	180	0	3	0	2
BC1415	PR	55	invasive lobular; multifocal	53	0	0	15	1	40	0	2
BC1141	PR	55	invasive lobular	52	1	1	500	0	20	0	2
BC1495	PR	60	invasive tubular a. intraductal (10%); bifocal	58	1	0	300	0	2	0	2
BC1497	PR	75	invasive lobular	42	1	1	300	0	5	2+	2
BC1160	PR	75	invasive lobular	47	0	1	30	0	35	0	2
BC1038	PR	75	invasive ductal	35	1	1	294	0	30	0	2
BC1095	PR	85	invasive tubular-lobular	60	1	0	294	0	1	0	1
BC1024	PR	88	invasive lobular	59	1	1	70	0	18	0	2
BC1101	PR	75–85	invasive lobular; multifocal	75	1	0	180	0	10	3+	2
BC1139	PR	89–90	invasive lobular with DCIS parts; multifocal	55	1	1	NA	0	15	NA	1

ER and PgR status were determined by immunohistochemistry. 1 – positive (>7 fmol/mg protein); 0 – negative (<7 fmol/mg protein). HER2/neu status: copies of HER2/neu gene. Status of p53 oncogene: 0 - <180 score; 1 - >180 score. NA – not available.

**Table 2:** Clinical and molecular data on breast cancer patients (test set).

Case	Response	Tumor reduction, %	Histology pre	Age	ER	PR	BCL2	P53	KI67, %	CerbB2	Grading
BC1843	NC	0	invasive ductal; bifocal	63	1	1	200	0	18	1+	2
BC1850	NC	0	invasive lobular	58	1	1	300	0	6	0	2
BC1862	NC	0	invasive lobular a. intraductal	59	1	1	196	0	11	1+	2
BC1871	NC	10	invasive lobular	46	1	1	140	1	24	0	2
BC1869	pCR	100	invasive ductal	60	0	0	0	0	50	0	3
BC1864	pCR	100	invasive a. intraductal (DCIS; 80%)	55	0	0	0	0	50	0	2
BC1421	pCR	100	invasive lobular	71	0	0	0	1	26	0	2
BC1870	cCR	100	invasive ductal	43	0	0	0	1	35	1+ to 2+	2
BC1861^1^	PR	40	invasive ductal a. intraductal (very small)	36	0	0	0	0	35	0	3
BC1879	PR	47	invasive ductal	37	1	1	500	0	45	0	2
BC1866	PR	40	invasive lobular	52	1	1	300	1	24	2+ to 3+	2
BC1837	PR	90	invasive ductal	69	0	0	0	0	48	1+	3
BC1838	PR	80	invasive lobular	59	1	0	50	0	20	1+ to 2+	2
BC1842	PR	92	invasive ductal	68	0	0	0	0	29	1+	2
BC1834	PR	0	invasive ductal a. intraductal (very small)	60	1	1	30	0	10	3+	3
BC1858	PR	0	invasive lobular	62	1	1	140	0	16	1+	2
BC1880	PR	40	invasive ductal and intraductal (5%)	62	1	1	200	0	26	0	2
BC1881	PR	62	invasive ductal	72	0	0	0	1	22	0	2
BC1849	PR	22	invasive ductal	52	1	0	200	0	19	3+	2
BC1839	PR	10	invasive ductal	62	0	0	45	1	16	0	2
BC1513	PR	33	invasive lobular; multicentr.	60	1	1	300	0	10	0	2
BC1877	PR/NC	50	left: invasive lobular a. CLIS Type-A	53	1	1	300	0	6	1+	2
BC1853	PR/NC	0	invasive lobular	51	1	1	400	0	14	0	2
BC1448	PR	68	medullary invasive	50	1	1	30	1	38	3+	3
BC1134	PR/NC	5	invasive lobular	73	1	0	70	0	14	0	2
BC1840^2^	PR	25	invasive ductal	45	1	1	60	0	35	2+ to 3+	3
BC1848	PR	85	invasive ductal; bifocal	42	1	0	500	1	28	3+	2

Note: 1 – This patient has received 4 × EC and, additionally, 4 × Taxol; 2 – this patient has received 3 × EC and, additionally, 3 × FEC.

### Immunohistochemistry (IHC)

Hematoxilin/eosin-stained sections from tumor specimens were examined to assess the relative amounts of tumor cells, benign epithelium, stroma, and lymphocytes. Standard clinical parameters, such as estrogen receptor-α (ER), progesterone receptor (PgR), proliferation marker (ki-67), tumor suppressor p53, regulator of apoptosis Bcl2, protooncogene cerbB2/HER2neu, epidermal growth factor receptor (EGFR) were assessed according to routine bio- and/or immunohistochemical methods.

Immunohistochemical staining was performed on 5-μm paraffin sections. Sections were deparaffinized in xylene and re-hydrated. Epitope retrieval was performed by heat induction in Target Retrival Solution pH6.1 (DAKO, DakoCytomation GmbH, Hamburg, Germany). Tissues were blocked for endogenous peroxidase in a 0.3% H_2_O_2 _solution for 15 min. Monoclonal antibodies (ERa: ER1D5 DAKO 1:35, PR: PGR636 DAKO 1:50, bcl-2: Clone 124 1:200 DAKO, EGFR: 31G7 CYTOMED 1:20, cerb2/Her-2/neu polyclonal DAKO 1:250, and ki67 (MIB-1) DAKO 1:200) were used for specific epitope detection. The ChemMate DAKO peroxidase/DAB Detection Kit was used for linking and staining. Slides were counterstained with methyl green and coverslipped with entelan. Histologic scores were calculated by multiplying color intensity (range 0 to 5) with proportion of cells staining positive.

### Response Criteria

Response to the treatment followed the Unio Internationale Contra Cancrum criteria [23]. pCR (pathological diagnosis based complete responders), was defined as absence of invasive carcinoma in the breast by the examining pathologist and lack of lymph nodal involvement. cCR (clinical complete responders) was defined as clinical absence of invasive carcinoma of the breast. This parameter was used as a surrogate for pCR in one occasion when a patient declined post-PST surgical excision. PR (partial responders) was determined as a reduction in the tumor mass of both perpendicular dimensions ranging from 10% to 75% of the initially measured tumor size based on dynamic contrast-enhanced magnetic resonance imaging or magnetic resonance tomography (MRT), and sometimes on both, MRT and ultrasonography. NC (non-responders or no change) was defined as an absence of tumor reduction or an increase in tumor size (stable or progressive disease). The percent of tumor reduction (Table 1 and 2) was calculated as a ratio between pathologic tumor size [cm] after neoadjuvant chemotherapy at the time of surgical excision compared to the size of tumors defined clinically at the time of diagnosis. More details are available as Additional files 3 and 4 from the JTM Web page.

### RNA Preparation and Microarray Analysis

Total RNA was extracted from cell lysates of ground tissue and subsequent purification with RNeasy mini spin columns (Qiagen, Hilden, Germany). Subsequent washing and elution steps were performed according to manufacturer's instructions. High-quality RNA was obtained as suggested by well-preserved 28S and 18S ribosomal RNA bands (present in an approximately 2:1 intensity ratio), along with A260/A280 ratios between 1.8 and 2.0. Quality and integrity of total RNA was tested with a Bioanalyzer 2100 (Agilent Technologies Inc., Palo Alto, CA, USA). Gene expression analysis was performed on an Affymetrix Human Genome U133A GeneChip platform containing 22,283 probes. Preparation and processing of labeled and fragmented cRNA targets, hybridization and scanning procedures were carried according to the manufacturer's protocol (Affymetrix, Santa Clara, CA, USA) [24]. Starting material for labeling consisted of 5 μg of total RNA from each tumor specimen. Labeling was limited to one cycle of *in vitro *transcription. Thus, starting with 5 μg of total RNA, approximately 50 to 60 μg of amplified RNA (cRNA) could be generated, which could be used in multiple microarray experiments. The cRNA was quantified by Agilent Nano Chip technology and evaluated for size relative to pure polyadenylated RNA. Fifteen micrograms of cRNA were subsequently used for hybridization. After washing and staining arrays were scanned by Gene Array scanner 2500 (Affymetrix). Hybridization intensity data were automatically acquired and processed by Affymetrix Microarray Suite 5.0 software. The expression level (average difference) of each gene was determined by calculating the average of differences in intensity (perfect match-mismatch) between its probe pairs as described elsewhere [25]. Scans were rejected if the scaling factor exceeded 2 or "chip surface scan" revealed scratches, specks or gradients affecting overall data quality (Refiner, GeneData AG, Basle, Switzerland).

### Quantitative Real-Time PCR.

Aliquots of total RNA used for GeneChip expression analysis were used for quantitative RT-PCR with an ABI PRISM 7900 Sequence Detection System (Applied Biosystems, Foster City, CA, USA). cDNA for PCR amplification was generated by oligo dT primed reverse transcription (Superscript First Strand System, Invitrogen Corporation, Carlsbad, CA, USA) including DNAse I treatment. Primers and probes were designed with the Primer Express software (Applied Biosystems, Foster City, CA, USA) and spanned the same gene region of the respective Affymetrix probe set. Labeled oligonucleotides were obtained from Eurogentec s.a. (Liege, Belgium). Absolute copy numbers were normalized according to *GAPDH *as a reference gene. The primer/probes were prepared by mixing 25 μl of 100 μM stock solution "upper primer", 25 μl of 100 μM stock solution "lower primer" plus 12,5 μl of the 100 μM stock solution TaqMan-probe (FAM/TAMRA) and adjusted to 500 μl with H_2_O (Primer/probe-mix). PCR reactions using cDNA generated from 1.5 ng total RNA were performed in duplicates in a volume of 10 μl. This included TaqMan universal Mix (Eurogentec s.a.) according to manufacturer's protocol in a 384-well format and 1 μl of the P&P mix. Thermal cycler parameters were 2 min at 50°C, 10 min at 95°C and 40 cycles, each consisting of a 15 s denaturation step at 95°C and a 1 min annealing/extension step at 60°C. Relative abundance of a gene transcript was calculated either by the ΔΔCt method or by arbitrarily defined RNA copy number estimates at a Ct = 24 as 10^6 ^copies. Subsequent analysis included normalization steps such as median centering and per gene median division.

### Data Filtering

Fifty-six primary breast cancer and 5 non cancerous breast tissue samples were analyzed as a training set for marker discovery. Raw data was acquired using Microsuite 5.0 software from Affymetrix and normalized following the standard practice of scaling the average of all gene signal intensities to a common arbitrary value (TGT = 100). Gene expression data were stored including *P*-value, as generated by Microsuite 5.0 software, for quality assessment of individual measurements for each transcript. The data-file was imported into Expressionist Analyst software package (GeneData AG) for further statistical analysis. To enhance quality we excluded gene probe sets for the following reasons. Fifty-nine probe sets corresponding to hybridization reference (housekeeping genes, *etc.*) as identified by Affymetrix were removed with the exception of *GAPDH *and β *-actin*, for which a 3' biased probe set was included. One hundred genes, whose expression levels are routinely used for normalization of the HG-U133A and HG-U133B GeneChip versions [26], were also removed from the analysis. These genes reflect a very homogenous expression pattern among several human tissues and could therefor be categorized as "house keeping" genes. Genes with potentially high levels of noise (81 probe sets) frequently observed with low absolute expression values (below 30 relative signal units (RSU) in all experiments) were also removed. The remaining genes were preprocessed to eliminate those (3,196) whose signal intensities were not significantly different (*P *> 0.04) from their background levels and thus labeled as "Absent" by MicroSuite 5.0. To apply a higher stringency to the data, we eliminated genes whose significance levels (*P *< 0.04) were only reached in 10% of the breast cancer samples (3,841 probe sets). Data for the remaining 15,006 probe sets were used for the subsequent analysis.

### Statistical Analysis

For the analysis we applied a similar strategy to the one applied by Wang E. and colleagues [27] to predict immune responsiveness of melanoma metastases. Genes differentially expressed by lesions characterized by different responsiveness to PST were identified with the nonparametric Wilcoxon rank sum test, two-sample independent Students't-test and Welch test. Probes were ranked in order of significance (SUM-Rank test) combining the results of these tests using as a cut-off *P-*value < 0.05 and fold change between groups >2. The Kruskal-Wallis and ANOVA tests were applied when two distinct groups (i.e. pCR *vs *NC) with extreme response patterns where studied in the presence of a third intermediate group (PR). All statistical tests were two-tailed. Principal components analysis (PCA) and hierarchical clustering were applied for data display and structural analysis and in certain steps for dimensional (probe set) reduction. All these different tools were used as implemented in the GeneData Expressionist Analyst software package and were only modified by selection of starting parameters and appropriate distance weight matrices. Additionally, partial least squares discriminant analysis for multivariate data (PLS-DA) with SIMCA-P software (Umetrics, AB, Umea, Sweden) was used.

## Results

### Preliminary analysis about ER status and inflammation

Previous studies [28-31] reported that patients with negative estrogen receptor (ER) status respond better to PST compared to those with a positive one. In addition, *PgR1 *gene expression may affect outcome. Furthermore, patients with ER-negative tumors suffer shorter disease-free and overall survival [32-34]. ER status is also associated with a characteristic gene expression profile independent of other clinical/pathological parameters [35,36]. Therefore, we separately studied genes known to be associated with ER signaling. We analyzed previously the expression profile of breast cancer in two patient cohorts with positive and negative ER status (not part of this study). The complete gene list and expression data within the two cohorts is available (Additional file 5). We identified 828 Affymetrix probe sets by ANOVA and *t*-test (*P *< 0.005) with a median fold change of 1.2 or above. Analysis of the 828 ER-related signatures in the 56 tumors from the present study correlated well with ER-α status by immunohistochemistry. To avoid the influence on clinical outcome of ER-specific signatures and identify alternative, ER-independent predictors of response and survival, these genes were excluded

We also excluded genes related to immune function since we could not predict the effect that the heterogeneity of immune infiltrates might bear on the transcriptional profile of individual lesions. Immune genes (1,025) were identified and excluded. The complete list of excluded genes is available (Additional file 6). Many of the excluded genes are members of immunoglobulin families. The final data set contained 13,145 probe sets. Although there is currently plenty of interest about the impact of immunity as a predictor of clinical outcome, this was beyond the purpose of this work and will be considered in a subsequent manuscript.

### Determination of predictor genes

Starting with the training cohort, we built response subclasses based on the post surgical clinicopathological examination. Eight of the 56 training cases experienced a pCR and eight progressed (NC). To identify the most predictive genes for each class we implemented a comparison schedule for the training set as follows:

(I) PR vs. NC (n = 40 vs. 8); (II) pCR vs. PR (n = 8 vs. 40), and (III) pCR vs. NC (n = 8 vs. 8). These comparisons were carried out by non-parametric *t-*test, Welch, Wilcoxon, Kolmogorov-Smirnov tests using the Expressionist Analyst software (GeneData AG). Differentially expressed genes were considered those reaching a significance cut off *P-value *of < 0.05 in all tests; 2,301 were identified. Additional restrictions were then applied (at least 2-fold change of median expression level and average expression more than 30 RSU (relative signal units) in all three groups) resulting in only 1,512 probe sets useful for further analyses.

For the "three-group tests" (pCR vs. PR vs. NC) statistical significance was measured with the Kruskal-Wallis and one-way ANOVA tests with a cut off *P-*value of < 0.05 identifying 414 probe sets. Overlap of the gene lists (1,512 probe sets and 414 probe sets) by Venn diagram analysis qualified 397 probe sets. This high stringency potentially eliminated genes of interest but decreased the false discovery rates of random selected genes at *P*-value cut off <0.05. PCA using all predefined tissue classes: non cancerous breast tissue pCR, cCR, PR and NC was applied to the 397 probe sets. Separation of pCR and cCR tumors on the one side and NC samples on the other was defined by 2 most distinguishing components. We applied a cutoff on the correlation matrix of the PCA and filtered genes at < -0.4 and > 0.4. This sorted out 325 by eliminating 72 probe sets.

We then excluded from the remaining 325 genes those known to be specifically expressed in blood vessels, adipocytes, and muscle tissues based on differential expression profiling of tumor cells and normal cells after their separation by laser capture microdissecction or by comparing breast tumor's gene expression profiles with expression profiles of normal blood vessels, adipose and muscle tissue samples reducing the number of genes by 61. The list of the excluded 61 genes is available (Additional file 7). Rank ordering of the remaining 264 genes' significance was determined by SUM-Rank test for all samples and compared to the original 13,145 genes.

In addition, two classifier genes were identified (*FHL1 *and *CLDN5*) highly discriminative between most "normal" tissue samples and all breast cancer samples analyzed. Whereas these genes are expressed at very high levels in normal breast tissue their low level expression was rarely detected in malignant breast samples. We combined these 2 genes with 57 most discriminative genes from 264 filtered probe sets (Additional file 8). Such combination allows simple and fast separation of normal tissue samples from malignant ones, which might be useful for routine clinical diagnostics. A detailed table containing raw data for 59 genes and 83 tumors is available as supplemental information (Additional files 9 and 10).

### Validation on independent cases

The determined classifiers could be subdivided into three categories: those genes/probe sets capable to distinguish between (a) normal breast and breast cancer tissues (2 genes, *FHL1 *and *CLDN5*), (b) pCR or cCR from unfavorable outcomes (PR or NC) (31 probe sets or "favorable response signature"), and (c) NC and PR (26 probe sets or "poor response signature"). We expected that both signatures, favorable and poor, would separate the two most extreme classes pCR and NC and effectively recognize the respective expression patterns. These classifiers were challenged against samples from an independent test cohort (n = 27; 4 pCR, 4 NC, 19 PR; see Table 2 or Additional file 2). Classification was performed by *k*-NN (*k *= 3) following a three step decision tree based on the 59 genes listed above. All 27 tumor samples were correctly qualified as cancerous tissues using the two-gene signature (*FHL1 *and *CLDN5*). Whit the "favorable response signature" a group of 8 tumor samples was classified as CR or PR. Finally, the rest of the tumors were classified as NC or PR by the "poor response signature". There were four potentially wrong classified cases. Results of classification for the test cohort are shown in Table 3. Summarized results of validation, as well as sensitivity, specificity positive and negative predictive values (PPV and NPV, respectively) for each class are shown in Table 4.

**Table 3:** Comparison of predicted and pathologic response in test set.

Case	Tumor reduction,%	Response, pathologic	Predicted response PCA cross validated by *k*-NN	Predicted response PLS-DA model 1	Predicted response PLS-DA model 2
BC1843	0	NC	NC	NC	PR
BC1850	0	NC	NC	NC	NC
BC1862	0	NC	NC	NC	PR
BC1871	10	NC	NC	NC	PR
BC1869	100	pCR	CR	CR	CR
BC1864	100	pCR	CR	CR	CR
BC1421	100	pCR	CR	CR	CR
BC1870	100	cCR	CR	CR	CR
BC1861	40	PR	PR	CR	CR
BC1879	47	PR	PR	NC	NC
BC1866	40	PR	PR	CR	CR
BC1837	90	PR	CR	CR	CR
BC1838	80	PR	PR	NC	NC
BC1842	92	PR	PR	PR	PR
BC1834	0	PR	PR	PR	CR
BC1858	0	PR	NC	NC	NC
BC1880	40	PR	PR	PR	PR
BC1881	62	PR	NC	PR	PR
BC1849	22	PR	NC	NC	PR
BC1839	10	PR	NC	NC	PR
BC1513	33	PR	PR	NC	PR
BC1877	50	PR	PR	NC	NC
BC1853	0	NC	NC	NC	NC
BC1448	68	PR	CR	CR	CR
BC1134	5	NC	NC	NC	NC
BC1840	25	PR	NC	NC	NC
BC1848	85	PR	CR	CR	CR

Note: complete pathologic response shown as CR when predicted.

**Table 4:** Summarized results of validation on the test cohort.

	Predicted response k-NN	Predicted response PLS-DA; model 1	Predicted response PLS-DA; model 2
predicted CR	7	9	10
other	20	18	17

predicted PR	9	4	9
other	18	23	18

predicted NC	11	14	8
other	16	13	19

Sensitivity CR	100	100	100
Specificity CR	87	78	74
PPV	57	44	40
NPV	100	100	100

Sensitivity PR	53	24	35
Specificity PR	100	100	100
PPV	100	100	67
NPV	56	43	39

Sensitivity NC	100	100	50
Specificity NC	76	62	76
PPV	55	43	38
NPV	100	100	84

### PCA and Hierarchical Clustering

**Figure 1:**
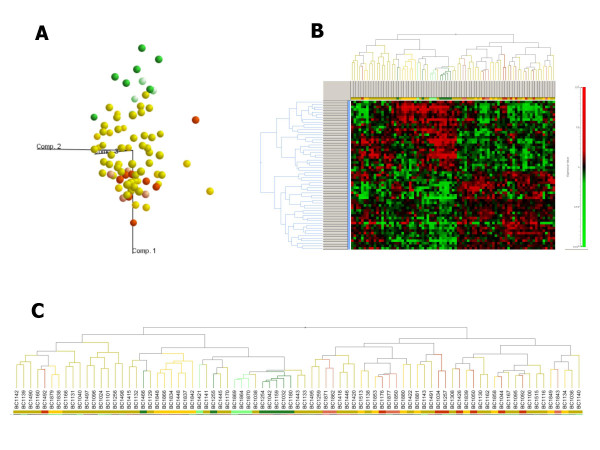
Clustering of gene expression data for 57 genes from 83 breast tumors corresponding training and test cohorts. A. PCA analysis of response groups and gene expression. The visualization of high-dimensional data in three-dimensional principal components. Individual samples from training and test cohorts are labeled according to three response groups: *green *and *light green *– pCR; *yellow *and *light yellow *– PR; *red *and *light red *– NC. The distance between samples reflects their approximate degree of correlation. B. Hierarchical clustering presents the clustered samples in columns and the clustered 57 genes in rows. A color representation of gene expression levels is shown with the scale on the *left *side. The 57 genes used fir both clustering methods were obtained by multi-step statistical approach, as described in 'A predictor gene set determination' section of Results. C. An enlarged version of sample dendrogram, which reflects similarities in their expression profiles.

A PCA plot was created displaying the position of each tumor sample from training and test cohorts (83 tumors) using three main Eigenvectors (Fig. 1A). The PCA was performed with the set of 57 response predictive genes for illustration purpose. The two most disparate response groups (pCR and NC) are clearly separated with the exception of one NC case, BC1492, which clusters with pCR tumors. This plot is consistent with *k*-NN cross-validation results for training cohort, which defined that NC case BC1492 as complete response. Hierarchical clustering of all 83 tumors and 57 response predicting genes is shown in Fig. 1B and 1C: eleven of twelve pCR tumors are organized in one sub-branch of the sample dendrogram and NC tumors are placed into the separate dendrogram branch.

### Partial least squares discriminant analysis (PLS-DA)

Direct linear discriminant analysis was applied to compare the previous results and test the potential of our first classifier model. PLS-DA applies well to the large number of predictors and the multicollineality. Supervised PLS-DA analysis uses independent (expression levels) and dependent variables (classes) for class comparison applying multivariate statistical methods such as soft independent modeling of class analogy (SIMCA) and partial least squares modeling with latent variables to allow simultaneous analysis of all variables [37-42]. Additionally, PLS-DA provides a quantitative estimation of the discriminatory power of each descriptor by means of *VIP *(variable importance for the projection) parameters. *VIP *values represent an appropriate quantitative statistical parameter ranking descriptors (gene expression values) according to their ability to discriminate different classes.

PLS-DA was carried out on the original 13,145 probe sets that passed the QC filtering process in the training cohort. Although this process may lead to an over parameterized model with poor prediction properties, it provides a preliminary assessment of the most important discriminative variables. Two independent models were tested each consisting of two classes: model 1 (class 1 – pCR, class 2 – NC, and PR cases were excluded); model 2 (class 1 – pCR, class 2 – NC and PR together). The model with three classes (pCR, NC and PR) demonstrated rather poor prediction power being strongly dependent on the definition of partial response (Table 1). Possibly the comparison of pathological estimates (post treatment) compared to clinical measurements (pre-treatment) over estimated the tumor reduction measurements and biased the attribution of samples as PR rather than NC.

**Figure 2:**
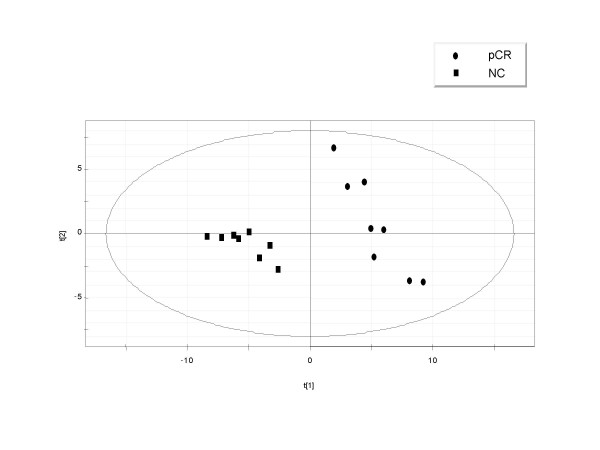
PLS discrimination according to tumor response class using the variables selected by PLS (*VIP *> 1.9) and ratio (pCR/NC) > 1.9 or < 0.55. Model 1 (PR cases were deleted; class 1 – pCR, *black dots*; class 2 – NC, *black squares*); 96 probe sets (cDNAs) retained.

**Figure 3:**
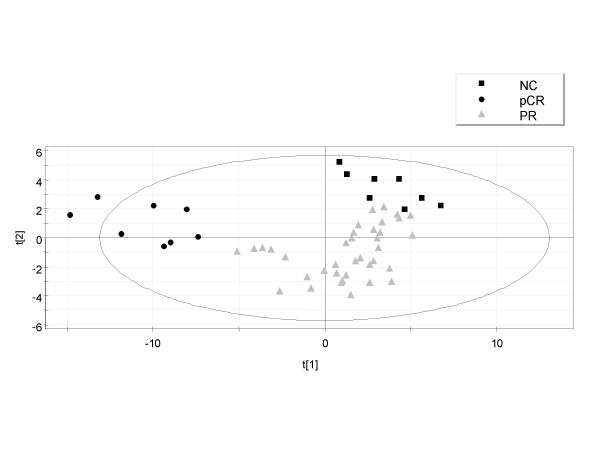
PLS discrimination according to tumor response class using the variables selected by PLS (*VIP *> 1.9) and ratio (pCR/NC) > 1.9 or < 0.55. Model 2 (class 1 – pCR, *black dots*; class 2 – NC, *black squares *and PR, *gray triangles*); 90 probe sets retained.

Those variables satisfying the criteria of expression levels above 60 RSU (as a mean value in at least one of each sample group, pCR and NC), ratio (pCR/NC) >1.9 or <0.55, and *VIP *of >1.9 were retained. Figure 2 shows a scatter plot of samples from the training set grouped according to the two components for either PLS in model 1 (96 probe sets; Fig. 2) or in the model 2 (90 probe sets; Fig. 3) after the second iteration. The numbers next to the symbols are the sample IDs as detailed in Table 1. It is apparent that pCR and NC samples are clearly discriminated. However, the results of permutation tests for both models (data not shown) demonstrated that both reduced models were still over-parameterized. Thus, we retained the 20 probe sets deduced from model 1 and 20 probe sets from model 2 with highest *VIP *values. In both cases, models performed much better than expected by chance.

Two groups of selected probe sets were compared and nine probe sets were found to be represented in both lists, which were deduced from model 1 and 2. A combined list containing 31 probe sets was used for model validation (Table 5) by applying PLS-DA to the second, independent group of tumors (n = 27; Table 2) to test the discriminative power of the final gene list. The results are presented in Table 3. PLS-DA classified partially responding tumors with good (> 60% tumor shrinkage) or very poor response to therapy as complete response (*e.g*., BC1837, BC1848, BC1448) or no response (*e.g.*, BC1877, BC1134, BC1840) respectively. This observation indicates that for further studies the monitoring of tumor shrinkage during PST is pivotal to correctly judge the final response classification and it might have been the major limitation of this study. Both statistical approaches, one that yielded the 59 gene and PLS-DA were compared and identified 19 genes in common. PLS-DA alone demonstrated a lower predictive power compared to the first multi-step analysis combined with *k*-NN classification.

**Table 5:** Top 31 genes extracted from two different models from PLS-DA SIMCA.

Gene Symbol	Gene Description	Ref. Sequences	Unigene ID
KPNA2	nuclear localization sequence receptor hSRP1alpha, karyopherin alpha 2 (RAG cohort 1 importin alpha 1)	NM_002266	4504896
*HDAC2*	transcriptional regulator homolog RPD3 histone deacetylase 2 similar to yeast RPD3	NM_001527	4557640
*PRKAB1*	5-AMP-activated protein kinase beta-1, non-catalytic subunit	NM_006253	18602783
*IMPDH2*	inosine monophosphate dehydrogenase (IMPDH2)	NM_000884	4504688
*YR-29*	hypothetical protein clone YR-29	NM_014886	7662676
*CD2BP2*	CD2 antigen (cytoplasmic tail)-binding protein 2	NM_006110	5174408
FHL2	heart protein (FHL-2) four and a half LIM domains 2	NM_001450	4503722
*DDB2*	damage-specific DNA binding protein p48 subunit (DDB2; 48 kD)	NM_000107	4557514
*ASNS*	asparagine synthetase	NM_001673	4502258
XPA	XPAC protein xeroderma pigmentosum complementation group A	NM_000380	4507936
*PLA2G7*	LDL-phospholipase A2 phospholipase A2 group VII (platelet-activating factor acetylhydrolase plasma)	NM_005084	4826883
*BTBD2*	BTB (POZ) domain containing 2 hypothetical protein FLJ20386 EST	NM_017797	8923361
*CCNG1*	cyclin G1 clone MGC:6	NM_004060	-
*PDHB*	pyruvate dehydrogenase E1-beta subunit d pyruvate dehydrogenase (lipoamide) beta	NM_000925	4505686
*MKI67*	mki67a (long type)antigen of monoclonal antibody Ki-67	NM_002417	4505188
*TNRC15*	KIAA0642 protein trinucleotide repeat containing 15	AL_045800	18550089
*RPL17*	ribosomal protein L17	NM_000985	14591906
*GNG12*	DKFZp586B0918 (from clone DKFZp586B0918)	NM_018841	-
*RPL17*	ribosomal protein L17	NM_000985	14591906
*DKC1*	Cbf5p homolog (CBF5) dyskeratosis congenita 1 dyskerin nucleolar protein	NM_001363	15011921
DCTN4	dynactin p62 subunit dynactin 4 (p62)	NM_016221	14733974
*FLJ20273*	RNA-binding protein	NM_019027	9506670
*FLJ11323*	hypothetical protein EST	NM_018390	8922994
*MGC11242*	hypothetical protein MGC11242 ESTs	NM_024320	13236560
*SRR*	serine racemase Homo sapiens cDNA	NM_021947	8922495
*ARL3*	48c8 ADP-ribosylation factor-like 3 EST	NM_004311	4757773
CCNB2	cyclin B2	NM_004701	10938017
*MAD2L1*	MAD2 protein MAD2 (mitotic arrest deficient yeast homolog)-like 1	NM_002358	6466452
*LIG1*	membrane glycoprotein LIG-1d	NM_015541	18554950
*PMSCL1*	polymyositisscleroderma autoantigen 1 (75 kD) EST	NM_005033	4826921
APBB2	amyloid beta (A4) precursor protein-binding, family B	NM_173075	18557629

Note:  – Genes in common with EC predictor (Table 3).

### Confirmation of expression measurements by real-time RT-PCR

**Figure 4:**
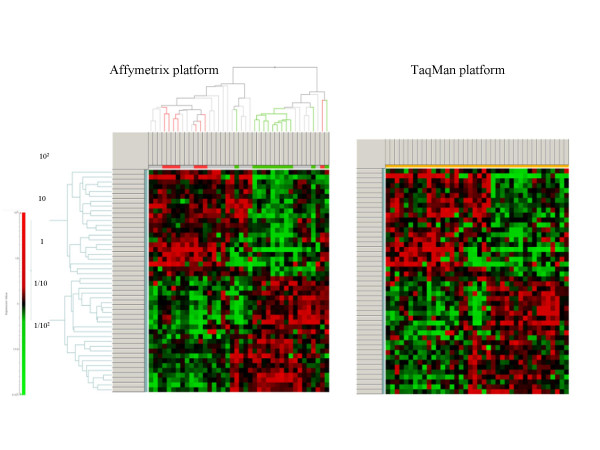
Confirmation of expression measurements by real-time RT-PCR. GeneChip median expression for 46 genes from Table 3 within one sample was adjusted to 100 RLU. Then, all measurements were median centered for each gene. Hierarchical clustering algorithm was applied to median normalized expression data of 46 genes from 39 tumor samples from training and test cohorts. Hierarchical clustering presents the clustered samples in columns and the clustered 46 genes in rows. A color representation of gene expression levels is shown with the scale on the *left *side (pCR represented in *green*, NC represented in *red*). Clustering of the data was performed according a correlation analysis with an average distance determination. The threshold Ct values obtained in real-time RT-PCR were converted into an arbitrary RNA-copy number Ct value of 24, which was then empirically settled to 10^6 ^RNA copies per well. These measurements were median centered, as for microarray data. All data for samples and genes were ordered according to the hierarchical structure of the microarray data set in Fig. 3A for Affymetrix platform.

**Figure 5:**
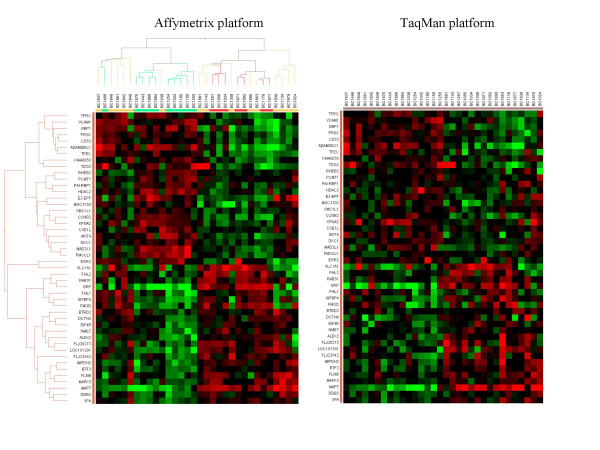
Confirmation of expression measurements by real-time RT-PCR Independent clustering of the qPCR data for both, Affymetrix and TaqMan platforms resulted in similar correlation trees.

Real-time RT-PCR (qPCR) measurement of gene expression levels on the same RNAs used for GeneChip hybridization experiments obtained from 32 breast tumors from training and test cohorts was performed on 46 genes selected from those presented in Table 3. Primer and probes were designed in regions within or close to the target region of the GeneChip oligonucleotides. A Ct value of 24 was empirically considered to represent 10^6 ^RNA copies per well based on spiking experiments. Raw data from real-time RT-PCR are presented in Supplemental Data on the Web page, as above, along with Affymetrix GeneChip's data. Relative expression as measured by the GeneChip was compared with qPCR results adjusting the median expression of all 46 genes within one sample to 100 relative units. To detect the relative difference in expression between samples for each gene, all measurements were divided by the median expression of this gene. This median normalization was carried out for both platforms independently. Raw and normalized data for Affymetrix and TaqMan platforms are shown in Additional file 11. In order to compare the individual measurements and the relative abundance of each transcript we preformed hierarchical clustering with the data generated with the GeneChip system. We performed this clustering (Fig. 4) with a correlation matrix on the samples as well as on the genes while the distance measurement was carried out with an average weight matrix. Once having the cluster of the GeneChip data in place we ordered all samples and all genes for the qPCR data in the same order as derived from the previous clustering. This operation resulted in very similar heat-maps as depicted in Figure 4 with an overall correlation of *R*^2 ^= 0.73. We also performed independent clustering of the qPCR data (Fig. 5), which resulted in similar correlation trees.

## Discussion

The aim of this study was to identify a multigene predictor of response to EC in a PST. Several recent studies demonstrated that gene expression profiling can predict response in the neoadjuvant setting [43-47]. Since the patient-specificity of such predictors remain questionable [48], further attempts devoted to the understanding of the process (es) underlying responsiveness to systemic therapy are of obvious importance.

Primary systemic chemotherapy is often being used to downstage large and locally advanced breast tumors in patients prior to surgery. There is increasing evidence that response and, particularly, complete response to neoadjuvant chemotherapy predicts improved disease-free and overall survival [49-51]. Unquestionable, pathological complete response (pCR) is not a synonym for cure, since a risk remains for metastatic disease. But such risk is decreased in association with the down-staging of the primary tumor and the achievement of a node negative status confirmed at the time of surgery. Therefore, it is reasonable to suggest that a good response to neoadjuvant therapy may correspond to survival benefit.

The role of biological characteristics and/or molecular markers as predictor of sensitivity to specific treatments has been extensively studied [52-56]. However, their role in response prediction remains unclear. Results from different studies are often contradictory and, consequently, no individual biological marker can be reliably used clinically for prediction of response to chemotherapy [57,58].

The patients analyzed in this study were part of a much larger cohort (n = 319) receiving treatment with EC-based PST. We have observed in this patient population that age, histologic grade, estrogen receptor (ER), progesterone receptor (PgR), levels of oncogene B-cell leukemia 2 (Bcl2), proliferation-related Ki-67 antigen (ki-67), and epidermal growth factor receptor (EGFR) expression were related to response in a univariate analysis, also confirmed by Colleoni *et al*. [33] in preoperative settings. However, in a multivariate model it was only ki-67 expression that predicted a better pathological response (*P *= 0.011), and this factor was linked to the patient's age [59]. Thus, a true predictive marker that could be measured by routine methods (*e.g*., IHC) to identify patients likely to benefit from neo-adjuvant EC remains elusive.

Several studies on breast cancer assessed classifiers predictive of survival [60-65]. A Dutch group reported 70 genes predictive of disease recurrence in women with lymph-node-negative primary breast cancer and confirmed the findings in a second study comprising additional 198 patients [65]. This study could assign some women to a low-risk category beyond the discriminating power of conventional histopathological criteria.

However, the concordance among different studies on survival of breast cancer patients is low. Data inconsistency can be particularly explained by the use of different microarray technologies and different patients' demographics. In addition, subtleties in data analysis may explain some discrepancies since there is no standardize method for expression data analysis when a large number of data points per individual are studied in relatively low sample populations.

In this study, we accurately discriminated samples that had a high tumor content from normal breast tissue based on the previous demonstration that *FHL1 *and *CLDN5 *can serve as such predictors. Then, we identified predictors in cancer tissues from primary tumors by identifying genes capable of segregating two distinct classes of tumors according to response to treatment (pCR *vs *NC). "Favorable outcome signature" could predict complete remission of a primary tumor with >90% sensitivity. Some genes found to be highly expressed in pCR samples belong to the "biological topic" of mitosis and cell proliferation (*e.g*. *MAD2L1*, *CCNB2*). This is concordant with the observations we [59] and others made on the *ki-67 *expression and the negative ER status in responding tumors [66]. Possibly, actively dividing tumors, either driven by the lack of hormonal control or by other signals such as via the insulin receptor pathway may respond best. The "poor outcome signature" distinguishing tumors unlikely to respond to PST included *DDB2 *or *XPA*, involved in DNA damage repair which makes perfect logical sense. The highest predictive value was sought in a stepwise manner by comparing pCR to NC cases and comparing predictors of each group by multi-step statistical approaches and *k*-NN (*k *= 3) validation. This classifier could predict with a remarkable level of accuracy a pathological response in the subsequent cohort of 27 patients used for validation. It is also possible that there were mis-assignments of responding cases especially in borderline cases that responded with minor changes in size or bifocal tumors. Ultra-sound imaging applied for size determination prior to chemotherapy might not be comparable to the accurate measurements that pathologists can make on resected samples. Thus, 10 cases in the training set considered as PR might not have qualified if comparable measurements could be used before and after therapy. This undefined error might have partially affect our statistical analysis decreasing the sensitivity of the model adopted (*i.e.*, predict many NC cases as PR and *vice versa*).

We also observed that application of different statistical algorithms to the data analysis lead to the extraction of overlapping predictor signatures (19 of 57 genes were in common). Although some of the genes identified by the PLS-DA could have been dismissed by the stringent filtering criteria applied, both analytical approaches could predict pCR. Accuracy of NC prediction could only be achieve through the stepwise identified signatures. Further in depth interpretation of the biological processes associated with the genes identified statistically will probably enhance the robustness of our findings in the future [67-69] [70].

We attempted to override the risk of overfitting of the model based on the training data (*i.e*., finding a mass of less relevant genes that may lead to the loss of a few relevant ones). The prediction accuracy was relatively high but was limited by the number of validation events (pCR or NC) so far analyzed suggesting that improved selection predictor genes among the ones identified based on a larger validation study may increase the accuracy of our findings and as a consequence their clinical value. We are currently collecting samples for a second validation cohort receiving EC based PST under similar conditions at an independent institution.

## Competing interests

The author(s) declare that they have no competing interests.

## Authors' contributions

OM and MM contributed equally to this work. All authors read and approved the final version of the manuscript.

## Supplementary Material

Additional File 1 contains clinico-/pathological information on patients in training cohort. Click here for file

Additional File 2 contains clinico-/pathological information on patients in test cohort. Click here for file

Additional File 3 contains more clinico-/pathological information on patients in training cohort. Click here for file

Additional File 4 contains more clinico-/pathological information on patients in test cohort. Click here for file

Additional File 5 contains the complete gene list and expression data within the two cohorts of patients with ER positive and ER negative status. Click here for file

Additional File 6 contains complete list of genes related to immune system (1,025), which were excluded from the analysis. Click here for file

Additional File 7 contains list of 61 genes. Those genes were excluded from the analysis by comparing breast tumor's gene expression profiles with expression profiles of normal blood vessels, adipose and muscle tissue samples. Click here for file

Additional File 8 contains EC predictor. Click here for file

Additional File 9 contains raw data for 59 genes and 83 tumors in training and test cohorts, respectively. Click here for file

Additional File 10 contains raw data for 59 genes and 83 tumors in training and test cohorts, respectively. Click here for file

Additional File 11 contains Affymetrix and TaqMan raw and normalized data for 46 genes and 32 primary breast tumors (confirmation study of expression measurements by real-time RT-PCR). Click here for file
