# Supplementary material for: Predictors of primary breast cancers responsiveness to preoperative Epirubicin/Cyclophosphamide-based chemotherapy: translation of microarray data into clinically useful predictive signatures
Source: J Transl Med. 2005 Aug 9;3:32. doi: 10.1186/1479-5876-3-32 (PMC1201176; doi:10.1186/1479-5876-3-32)
Supplement: Additional File 8 — contains EC predictor. [file 1479-5876-3-32-S8.doc]

EC Gene predictor.

| Group  Nr. | Gene Symbol | Gene Description | Cellular Localization | Biological Function | Ref. Sequences | Unigene ID |
| --- | --- | --- | --- | --- | --- | --- |
| NB | FHL1 | four and a half LIM domains 1 | intracellular | unknown | NM_001449 | 239069 |
| NB | *CLDN5* | transmembrane protein claudin 5 | membrane | tight junction; integral membrane protein | NM_003277 | 110903 |
| 1 | *CCNB2* | cyclin B2 | intracellular | cell cycle regulator | NM_004701 | 194698 |
| 1 | *E2-EPF* | ubiquitin carrier protein | intracellular | protein modification | NM_014501 | 174070 |
| 1 | *MAD2L1* | MAD2-like 1 | intracellular | mitotic cycle control; kinetochore | NM_002358 | 79078 |
| 1 | *PMSCL1* | polymyositis/scleroderma autoantigen 1, 75kDa | intracellular | nucleolus; nucleus | NM_005033 | 91728 |
| 1 | *KPNA2* | karyopherin alpha 2 | intracellular | mitotic G phase; DNA metabolism; regulation of DNA recombination; NLS-bearing substrate-nucleus import; spindle pole body and microtubule cycle | NM_002266 | 159557 |
| 1 | *PAI-RBP1* | PAI-1 mRNA-binding protein | unknown | unknown | NM_015640 | 165998 |
| 1 | *HDAC2* | histone deacetylase 2 | intracellular | transcription regulation | NM_001527 | 3352 |
| 1 | *KIAA0056* | - | unknown | unknown | - | 13421 |
| 1 | *RAB31* | RAB31, member RAS oncogene family | intracellular | non-selective vesicle transport | NM_006868 | 223025 |
| 1 | *FLJ20273* | RNA-binding protein | intracellular | unknown | NM_019027 | 95549 |
| 1 | *GRP* | gastrin-releasing peptide | secreted | signal transduction | NM_002091 | 1473 |
| 1 | *IMPDH2* | IMP (inosine monophosphate) dehydrogenase 2 | intracellular | IMP dehydrogenase | NM_000884 | 75432 |
| 1 | *FHL2* | four and a half LIM domains 2 | intracellular | oncogenesis | NM_001450 | 8302 |
| 1 | *DCTN4* | dynactin 4 (p62) | unknown | unknown | NM_016221 | 180952 |
| 1 | *DDB2* | damage-specific DNA binding protein 2 (48kD) | intracellular | DNA repair; pyrimidine-dimer repair, DNA damage excision | NM_000107 | 77602 |
| 1 | *YR-29* | TGF beta-inducible nuclear protein 1 | unknown | unknown | NM_014886 | 8170 |
| 1 | *IGFBP4* | insulin-like growth factor binding protein 4 | secreted | signal transduction; cell proliferation; DNA metabolism; skeletal development | NM_001552 | 1516 |
| 1 | *PLA2G7* | phospholipase A2, group VII (platelet-activating factor acetylhydrolase, plasma) | secreted | lipid metabolism; inflammatory response | NM_005084 | 93304 |
| 1 | *LIG1* | leucine-rich repeats and immunoglobulin-like domains 1 | secreted | unknown | NM_015541 | 4193 |
| 1 | *APBB2* | amyloid beta A4 precursor protein-binding, family B, member 2 | intracellular | signal transduction; protein phosphorylation | NM_173075 | 15740 |
| 1 | *VCAM1* | vascular cell adhesion molecule 1 isoform a precursor | membrane | cell cdhesion | NM_001078 | 109225 |
| 1 | *MAPT* | microtubule-associated protein tau isoform 2 | membrane | microtubule cytoskeleton organization and biogenesis; apoptosis | NM_005910 | 101174 |
| 1 | *EGR2* | early growth response 2 protein | intracellular | cell growth | NM_000399 | 1395 |
| 1 | *ADAMDEC1* | disintegrin protease | secreted | integrin receptor signalling | NM_014479 | 145296 |
| 1 | *BTF3* | basic transcription factor 3 | intracellular | transcription from Pol II promoter | NM_001207 | 101025 |
| 1 | *FLNB* | filamin B, beta (actin binding protein 278) | intracellular | cytoskeletal anchoring; signal transduction; actin cytoskeleton reorganization | NM_001457 | 81008 |
| 1 | *TFRC* | transferrin receptor (p90, CD71) | unknown | iron homeostasis; iron transport | NM_003234 | 77356 |
| 1 | *TMEM30B* | transmembrane protein 30B | unknown | unknown | - | 85335 |
| 1 | *SLC1A1* | solute carrier family 1, member 1 | membrane | glutamate transport; synaptic transmission | NM_004170 | 91139 |
| 1 | *BLAME* | we17h07.x1 BCM-like membrane protein precursor EST | intracellular | unknown | NM_014036 | 20450 |
| 1 | *FMOD* | fibromodulin precursor | secreted | TGFbeta receptor complex assembly | NM_002023 | 230 |
| 2 | *DKC1* | dyskerin | intracellular | cell cycle control; telomere maintenance; RNA processing; rRNA processing; cell proliferation | NM_001363 | 4747 |
| 2 | *CSE1L* | CSE1 chromosome segregation 1-like protein isoform α | intracellular | cell proliferation; nucleocytoplasmic transport; apoptosis | NM_001316 | 90073 |
| 2 | *LRFN4* | hypothetical protein MGC3103 | membrane | unknown | NM_024036 | 115960 |
| 2 | *RHEB2* | Ras homolog enriched in brain 2 | intracellular | signal transduction | NM_005614 | 279903 |
| 2 | *PCMT1* | protein-L-isoaspartate (D-aspartate) O-methyltransferase | intracellular | protein methylation | NM_005389 | 79137 |
| 2 | *AD-017* | glycosyltransferase AD-017 | intracellular | unknown | NM_018446 | 283737 |
| 2 | *FLJ22642* | FLJ22642 fis clone HSI06970 EST | unknown | unknown | - | 288232 |
| 2 | *SSR1* | signal sequence receptor, alpha | membrane | positive control of cell proliferation; co-translational membrane targeting | NM_003144 | 250773 |
| 2 | *SMC1L1* | SMC1 structural maintenance of chromosomes 1-like 1 | unknown | mitosis; mitotic chromosome segregation | NM_006306 | 211602 |
| 2 | *ARL3* | ADP-ribosylation factor-like 3 | intracellular | GTP binding | NM_004311 | 182215 |
| 2 | *SEMA3C* | semaphorin 3C | secreted | cell growth and maintenance; immune response; drug resistance | NM_006379 | 171921 |
| 2 | *XPA* | xeroderma pigmentosum, compl. group A | intracellular | DNA repair; nucleotide-excision repair | NM_000380 | 192803 |
| 2 | *BTBD2* | BTB (POZ) domain containing 2 | unknown | unknown | NM_017797 | 25817 |
| 2 | *TNRC15* | KIAA0642 protein trinucleotide repeat containing 15 | unknown | unknown | - | 323317 |
| 2 | *RPL17* | ribosomal protein L17 | secreted | RNA binding; structural protein of ribosome | NM_000985 | 82202 |
| 2 | *FLJ13125* | FLJ13125 fis clone NT2RP3002877 | secreted | unknown | - | 287551 |
| 2 | *PRG1* | proteoglycan 1, secretory granule | secreted | proteoglycan | NM_002727 | 1908 |
| 2 | *GBP1* | guanylate binding protein 1, interferon-inducible, 67kD | secreted | unknown | NM_002053 | 62661 |
| 2 | *ALEX2* |  | unknown | unknown | NM_014782 | 48924 |
| 2 | *CD53* | CD53 antigen | membrane | signal transduction; antimicrobial humoral response | NM_000560 | 82212 |
| 2 | *TDO2* | tryptophan 2,3-dioxygenase | unknown | neurotransmitter synthesis and storage | NM_005651 | 183671 |
| 2 | *TFEC* | transcription factor EC | intracellular | transcription co-activator; transcription co-repressor; transcription factor | NM_012252 | 113274 |
| 2 | *EIF4B* | eukaryotic translation initiation factor 4B | intracellular | translational regulation, initiation | NM_001417 | 93379 |
| 2 | *MAPK3* | HSERK1 ERK1 protein serine threonine kinase | intracellular | signal transduction; chemotaxis | - | 861 |
| 2 | *MST4* | serine/threonine protein kinase MASK | intracellular | unknown | NM_016542 | 23643 |
| 2 | *NME7* | NME7 | intracellular | unknown | NM_013330 | 274479 |
