# Supplementary material for: Predictors of primary breast cancers responsiveness to preoperative Epirubicin/Cyclophosphamide-based chemotherapy: translation of microarray data into clinically useful predictive signatures
Source: J Transl Med. 2005 Aug 9;3:32. doi: 10.1186/1479-5876-3-32 (PMC1201176; doi:10.1186/1479-5876-3-32)
Supplement: Additional File 9 — contains raw data for 59 genes and 83 tumors in training and test cohorts, respectively. [file 1479-5876-3-32-S9.pdf]

Gene	Locus_Link	BC1492	BC1426	BC1257	BC1176	BC1092	BC1050	BC1034	BC1044	BC1466	BC1255	BC1254	BC1180	BC1159	BC1042	BC1032	BC1443	BC1167	BC1162	BC1143
CCNB2	9133	289	33.56	112.18	44.09	63.41	82.82	75.23	37.52	94.14	115.64	329.41	223.81	215.8	247.65	277.91	280.09	40.19	46.83	49.42
E2-EPF	27338	594.51	449.44	193.86	99.91	196.81	197.87	208.1	128.08	87.6	436	680.14	346.68	529.66	322.13	754.52	503.95	81.85	103.5	186.37
MAD2L1	4085	198.08	66.24	53.09	47.58	40.98	89.35	50.84	21.24	33.43	79.4	186.85	176.7	665.12	278.17	159.84	256.84	34.83	26.64	42.77
DKC1	1736	513.49	442.71	305.66	247.42	295.83	285.11	322.8	223.69	334.78	603.84	877.7	394.53	989.47	711.32	470.22	441.55	244.54	301.12	203.62
PMSCL1	5393	105.13	22.85	42.68	20.2	30.66	45.69	23.81	1.43	21.83	39.69	156.51	121.41	262.35	166.99	77.57	106.88	23.05	30.53	33.9
KPNA2	3838	622.45	154.27	270.81	297.39	307.6	220.42	144.81	65.01	299.21	389.34	604.03	511.52	467.16	518.33	532.77	1130.72	128.11	219.65	111.84
CSE1L	1434	313.85	184.57	141.03	97.4	158.34	206.49	129.33	92.64	147.05	190.48	402.93	217.73	368.52	287.91	263.71	360.41	90.36	134.47	84
MGC3103	78999	120.18	30.13	41.41	24.44	20.54	28.24	10.17	6.29	84.17	70.73	52.77	66.01	112.91	73.08	93.37	60.26	26.52	62.6	40.26
PAI-RBP1	26135	1001.42	552.46	323.8	229.68	237.39	217.67	299.9	180.99	531.8	541.5	1076.03	725.09	1044.2	757.39	345.39	671.91	317.3	251.4	272.21
RHEB2	6009	475.87	308.78	260.28	214.42	265.03	191.8	176.44	86.22	186.71	499.38	495.14	436.23	555.04	441.75	347.03	299.84	268.95	234.33	192.59
HDAC2	3066	333.43	248.46	65.75	103.97	221.11	116.84	107.11	49.81	210.77	399.31	500.64	544.53	293.65	306.67	259.88	420.35	197.72	152.33	100.17
PCMT1	5110	374.33	419.28	150.43	188.37	228.94	187.58	129.59	154.35	265.5	637.84	444.51	362.31	510	263.2	470.19	445.25	388.5	180.78	224.26
KIAA0056	23310	153.5	195.7	31.9	39.3	43	74.36	53.13	46.5	77.6	99.37	90.38	110.57	162.85	116.33	76.67	88.68	27.44	49.58	71.85
RAB31	11031	67.77	108.71	835.77	726.86	905.12	448.45	342.09	1009.54	410.37	352.47	169.41	213.57	131.23	257.32	421.83	276.74	557.69	171.63	562.32
FLJ20273	54502	268.06	707.86	700.69	414.58	462.51	971.56	619.8	446.87	212.16	205.8	212.38	175.33	317.34	241.77	319.37	459.54	391.21	616.96	524.68
GRP	2922	4.4	25.89	66.46	148.43	168.08	70.73	65.28	153.91	37.95	38.33	2.76	5.65	4.72	16.26	4.56	18.53	156.34	3.92	125.25
AD-017	55830	257.23	351.88	257.81	262.22	290	265.32	187.68	210.11	174.13	183.7	145.54	171.77	131.03	87.56	150.19	166.75	407.36	264.97	249.43
IMPDH2	3615	549.5	1010.47	1002.19	808.5	1034.6	991.38	1027.16	701.1	309.89	539.98	497.79	414.2	340.86	196.2	384.84	543.58	560.51	919.59	753.01
FHL2	2274	122.05	238.93	522.3	714.07	610.92	429.17	499.67	202.78	314.67	149.61	241.18	213.95	114.39	130.92	75.67	293.42	288.37	280.17	513.49
FLJ22642	0	187.73	194.25	176.57	241.82	221.67	154.56	189.9	180.69	261.02	107.37	69.68	64.91	85.78	97.88	171.6	129.81	221.66	132.46	329.32
DCTN4	51164	81.52	210.07	120.37	153.46	69.44	99.72	112.84	118.73	86.58	71.31	61.42	46.18	69.03	50.75	51.34	100.97	119.71	101.99	98.18
DDB2	1643	17.31	37.97	98.13	46.83	63.01	86.77	42.43	77.04	21.76	47.73	17.08	30.8	27.49	30.86	28.41	24.33	105.34	72.95	56.31
YR-29	10412	408.77	1044.74	469.64	559.29	665.65	470.96	519.62	602.97	586.14	257.68	239.7	332.53	272.6	250.83	333.58	375.38	740.97	829.84	579.72
SSR1	6745	621.68	669.75	489.53	563.61	429.95	404.29	622.23	503.46	876.45	842.06	1367.04	681.67	850.48	778.67	668.84	956.66	532.08	614.07	412.32
IGFBP4	3487	110.27	543.01	1706.77	1898.7	1289.75	1090.63	1031.11	1632.16	1834.74	598.94	363.49	451.52	308.79	399.08	277.22	720.79	2364.87	1510.07	1409.62
SMC1L1	8243	256.91	211.8	127.62	208.44	139.98	270.39	174.55	119.65	273.25	210.14	344.97	364.64	286.03	422.89	355.92	461.74	152.29	253.3	90.02
ARL3	403	121.11	323.76	308.53	195.35	220.85	259.49	225.54	203.26	106.86	146.71	105.92	119.39	91.98	98.23	167.62	179.01	242.5	240.29	278.51
SEMA3C	10512	53.94	62	70.16	76.55	76.86	68.77	82.59	107.6	69.3	41.98	31.5	45.83	37.95	33.19	30.35	56.76	66.45	47.06	55.49
XPA	7507	62.1	90.94	132.79	81.93	97.06	92.91	91.95	79.64	62.81	61.63	36.6	58.37	64.79	47.7	30.31	35.67	73.13	67.61	69.74
PLA2G7	7941	64.7	18	38.79	22.52	18.96	26.3	30.66	11.76	47.25	119.76	138.83	188.91	94.31	109.37	62.7	79.25	4.31	33.44	29.27
BTBD2	55643	164.45	122.95	115.59	153.71	96.26	110.86	88.39	122.89	77.46	72.3	83.27	42.24	85.78	77.36	55.32	86.93	169.32	145.07	98.58
LIG1	26018	191.7	581.13	351.57	506.53	415.26	314.38	279.75	383.67	196.97	152.71	119.24	94.9	117.55	114.38	142.04	179.6	435.87	417.02	203.96
TNRC15	26058	116.25	120.24	99.72	121.9	120.4	119.87	121.1	125.36	101.77	57.99	63.98	81.68	72.69	93.92	84.2	117.67	103.24	80.25	69.09
RPL17	6139	2866.05	4178.35	2608.74	2535.36	2362.89	3313.09	3729.45	3001.2	1826.71	1436.54	1426.03	1922.21	1818.6	2103.24	2238.19	1824.74	2826.1	2793.01	4012.79
FLJ13125	0	27.3	44.73	39.75	33.08	40.64	32.29	27.39	31.64	45.21	54.12	43.28	80.18	48.46	57.57	81.69	49.67	48.5	30.48	43.55
APBB2	323	35.38	48.58	46.95	61.11	55.29	61.29	82.54	101.93	22.31	20.25	10.71	19.19	25.21	14.23	32.27	49.6	36.51	38.9	71.35
PRG1	5552	269.09	310.89	336.19	280.73	261.66	205.21	372.57	157.83	473.74	639.51	643.67	333.23	677.21	420.02	463.66	668.42	244.75	290.54	146.14
GBP1	2633	30.5	36.01	137.87	33.58	48.89	79.69	167.93	49.31	142.8	286.68	303.61	95.74	169.49	295.97	85.73	171.06	41.81	78.11	19.82
ALEX2	9823	288.69	326.94	234.83	310.96	292.23	279.41	264.04	203.02	124.71	36.82	97.17	91.97	151.94	63.17	161.11	151.58	226.68	328.68	146.31
CD53	963	211.4	198.09	486.71	342.8	446.59	359.94	594.03	191.06	707.01	688.78	688.65	671.85	583.25	603.87	460.7	608.84	281.36	338.87	192.25
VCAM1	7412	44.59	77.16	144.63	56.09	206.63	91.42	117.1	173.56	271.48	573.77	244.58	276.17	510.69	289.94	164.96	148.68	158.21	207.19	111.06
MAPT	4137	48.74	84.92	151.53	64.68	58.8	206.31	170.98	166.52	22.22	59.82	13.77	35.33	22.75	28.43	44.47	5.12	65.34	94.95	96.86
EGR2	1959	30.03	47.66	19.47	41.07	55.13	37.27	41.31	23.91	71.45	55.59	94.27	164.76	47.39	83.44	78	60.95	23.42	33.69	16.36
TDO2	6999	24.14	22.61	42.89	6.01	40.95	21.16	28.74	23.1	912.33	224.86	78.25	59.06	57.04	53.2	40.82	42.08	25.72	14.14	427.83
ADAMDEC1	27299	41.52	26.43	105.39	22.41	23.73	36.26	134.14	10.37	233.69	405.84	289.87	73.81	149.63	146.43	139.94	58.19	12.36	31.79	56.32
TFEC	22797	22.66	24.11	51.15	17.57	23.43	19.28	42.89	2.26	84.33	75.94	64.94	52.8	54.87	52.29	40.57	53.95	10.82	20.17	14.69
BTF3	689	1099.1	2095.24	2081.53	1620.91	1540.51	1901.68	1866.81	1873.69	931.81	709.45	1075.13	1061.89	978.9	862.35	1426.42	1090.18	1913.86	2058.85	2206.9
FLNB	2317	463.08	313.91	404.83	216.51	195.6	541.76	365.05	342	38.35	181.54	121.72	80.54	213.93	106.14	80.12	231.93	335	277.74	319.09
TFRC	7037	571.98	325.17	308.81	276.86	618.49	732.26	286.81	490.27	965.71	1174.48	1091.31	541.39	895.42	682.33	1050.78	852.92	524.07	412.27	466.6
EIF4B	1975	519.34	1018.01	475.47	611.89	673.01	558.77	1034.28	529.42	373.29	344.23	221.27	225.05	316.16	413.88	375.84	542.52	411.91	659.88	605.08
MAPK3	5595	288.58	157.44	279.74	234.77	151.52	345.75	116.9	220.05	61.14	83.82	100.2	91.14	68.93	83.13	130.94	127.3	489.24	194.68	145.39
LOC161291	161291	99.44	343.93	186.75	244.98	162.93	266.3	124.12	198.72	137.75	14.41	32.49	53.31	67.35	98.01	75.07	58	186.54	268.15	188.55
SLC1A1	6505	152.84	334.59	144.14	91.87	389.11	142.45	809.14	192.4	30.57	32.03	19.14	60.27	23.77	44.48	46.48	19.47	140.28	109.53	47.1
MST4	51765	31.01	12.76	44.71	29.11	35.38	33	44.55	23.71	54.39	153.79	241.81	77.47	84.88	173.73	82.31	66.95	89.3	50.42	42.91
BLAME	56833	20.24	14.12	85.31	21.35	25.16	50.56	82.81	3.85	84.93	234.1	136.23	42.39	109.1	103.95	101.71	71.32	19.03	28.42	30.94
NME7	29922	188.81	83.14	92.38	88.88	101.62	159.59	225.87	115.15	70.45	50.43	36.17	61.59	15.87	61.48	63.47	71.58	89.72	92.98	48.6
FHL1	2273	4.94	34.21	46.68	103.52	71.11	43.86	33.5	60.27	79	13.15	11.77	28.36	1.93	25.53	17.03	42.89	48.6	70.49	39.15
FMOD	2331	73.16	304.87	248.07	597.58	423.29	235.73	612.63	269.94	380.49	201.65	91.9	220.11	61.65	103.9	60.5	133.48	364.6	239.13	596.31
CLDN5	7122	7.49	99.35	11.79	49.76	55.43	30.4	10.65	52.2	65.06	26.22	6.48	60.34	6.28	18.38	12.22	11.71	59.64	65.48	26.79

Gene	Locus_Link	BC1138	BC1100	BC1040	BC1170	BC1140	BC1418	BC1420	BC1491	BC1515	BC1445	BC1036	BC1308	BC1133	BC1259	BC1498	BJ_40613	BC1166	BC1142	BC1422
CCNB2	9133	75	84.81	147.36	199.14	141.49	55.41	43.91	60.92	82.33	81.59	66.24	186.75	309.12	25.76	133.85	69.41	172.29	71.78	109.85
E2-EPF	27338	190.56	111.33	187.83	263.75	256.59	104.63	82.36	117.32	89.44	129.53	171.2	271.97	304.75	57.83	283.08	68.76	256.89	352.68	153.7
MAD2L1	4085	47.98	48.4	202.11	98.18	79.36	39.17	36.97	33.83	21.14	67.84	40.14	95.12	188	38.28	99.1	50.35	165.66	46.49	33.44
DKC1	1736	269.93	393.8	435.57	395.07	297.7	268.34	312.8	365.07	370.71	452.73	347.66	253.82	779.9	216.4	454.46	305.64	339.91	389.11	376.12
PMSCL1	5393	56.2	54.21	69.5	97.57	67.87	48.43	30.21	31.98	32.94	44.52	29.45	63.31	123.65	23.82	86.07	65.99	79.13	31.97	30.37
KPNA2	3838	180.21	194.97	430.06	257.45	328.16	289.98	216.81	222.26	298.49	263.96	196.51	302.33	444.38	224.92	363.4	240.47	368.73	373.61	101.27
CSE1L	1434	255.42	209.32	227.54	95.88	190.49	121.64	144.76	106.58	168.07	170.53	143.35	194.35	127.77	116.1	195.75	288.53	396.75	179.1	154.86
MGC3103	78999	75.31	36.4	35.44	75.44	47.59	40.61	26.82	61.83	38.5	68.59	52.03	3.05	76.82	4.88	93.6	31.74	158.69	61.73	34.58
PAI-RBP1	26135	216.86	462.01	417.11	274.94	447.9	422.79	392.63	308.79	318.93	454.81	247.29	702.12	1009.94	217.39	392.81	209.18	537.22	526.12	208.78
RHEB2	6009	195.12	252.71	379.11	165.5	314.11	331.3	200.73	247.29	208.17	281.12	257.86	284.08	299.53	175.14	356	152.2	297.51	196.69	292.58
HDAC2	3066	85.73	133.03	137.16	189.52	162.18	221.32	80.7	134.22	71.57	258.39	63.84	184.37	204.01	165.29	521.65	202.2	236.78	201.66	185.47
PCMT1	5110	211.78	256.32	239.99	917.97	328.6	355.54	232.55	146.03	221.24	299.39	132.29	243.66	242.15	407.09	820.89	287.43	245.2	274.81	180.79
KIAA0056	23310	14.02	64.75	54.22	4.88	53.94	26.03	49.67	58.98	47.98	25.37	38.35	75.85	82.1	2.31	59.51	45.78	45.58	56.23	29.12
RAB31	11031	630.23	788.66	647.67	885.43	716.77	832.4	630.99	820.93	1184.75	173.56	794.39	248.97	198.24	289.03	284.8	1933.13	427.58	516.05	1313.66
FLJ20273	54502	251.06	786.87	251.23	336.93	212.88	333.42	550.94	296.44	478.63	550.05	441.72	1218.78	120.03	458.86	660.97	325.38	500.89	364.89	413.61
GRP	2922	127.16	214.77	93.1	33.72	83.78	271.3	131.94	147.92	175.37	9.51	153.82	52.71	17.74	96.84	102.99	66.82	83.36	59.55	197.71
AD-017	55830	209.03	256.38	143.23	238.04	171.84	372.72	332.52	184.89	259.56	191.02	313.41	167.02	143.84	275.48	129.59	269.3	170.63	153.72	401.68
IMPDH2	3615	946.85	1228.32	599.82	480.89	550	489.95	696.99	573.57	980.05	511.99	648.91	1434.04	789.12	505.98	376.45	1172.46	592.95	1479.75	787.34
FHL2	2274	314.53	702.4	227.2	235.02	165.06	451.79	664.37	125.15	403.72	150.4	92.94	110.13	295.74	227.41	115.47	165.25	227.66	669.8	396.76
FLJ22642	0	259.33	230.85	144.08	111.67	181.69	193.22	218.39	130.68	201.98	96.68	293.49	762.12	68.19	259.9	184.7	235.99	221.53	119.72	353.26
DCTN4	51164	85.45	101.39	99	49.15	65.55	72.33	126.43	113.4	116.38	51.13	127.16	205.13	79.02	113.99	75.33	56.72	135.02	53.43	104.77
DDB2	1643	108.67	120.65	89.36	50.18	135.2	81.89	53.74	118.23	82.97	59.88	112.93	44.33	24.4	60.46	11.02	71.55	93.08	49.15	61.52
YR-29	10412	703.73	726.77	420.31	208.19	651.89	622.13	693.76	632.31	740.41	393.8	678.24	1050.47	611.17	574.32	332.88	471.44	484.92	865.25	494.99
SSR1	6745	597.72	563.66	414.91	727.4	819.05	743.72	554.1	510.6	405.36	1015.79	539.58	640.93	657.07	488.75	631.07	877.72	875.49	1383.94	436.45
IGFBP4	3487	2360.04	1032.14	1324.6	1143.82	3139.45	1781.06	1427.81	1643.26	1572.78	588.91	2477.44	250.47	590.62	847.97	561.43	728.73	1511.24	2561.37	1853.03
SMC1L1	8243	246.95	210.99	366.07	231.43	268.2	202.73	242.8	132.51	164.86	282.96	244.26	278.92	279.85	268.07	242.3	235.96	257.2	279.6	258.58
ARL3	403	186.74	222.82	352.92	131.67	234.56	261.93	175.65	169.37	284.83	173.26	222.27	99.16	129.01	215.96	141.77	157.6	239.84	256.8	203.64
SEMA3C	10512	84.21	64.3	67.33	42.62	42.95	40.9	63.06	67.42	45.49	69.8	44.52	181.21	27.37	40.36	57.13	60.03	46.83	33.32	105.28
XPA	7507	68.42	96.2	59.62	67.72	69.91	57.98	97.66	90.27	85.84	98.01	73.63	116.83	64.69	117.13	72.6	91.02	67.15	65.91	47.51
PLA2G7	7941	19.41	17.2	52.97	110.53	103.41	126.51	86.67	48.01	19.64	36.23	10.62	39.56	138.39	29.46	41.76	25.19	42.5	53.81	19.64
BTBD2	55643	87.71	102.95	40.84	97.94	109.58	53.03	86.65	64.39	124.71	90.06	230.57	77.82	209.15	45.76	75.92	60.53	100.28	139.04	144.26
LIG1	26018	239.52	352.56	137.3	196.3	314.25	115.44	352.51	155.66	431.44	285.47	201.6	485.55	150.25	357.19	146.27	224.83	304.7	314.83	199.87
TNRC15	26058	94.74	114.24	100.08	29.55	69.13	71.8	104.91	35.99	55.63	107.76	129.37	110.6	58.03	69.48	69.21	83.86	84.45	89.95	105.86
RPL17	6139	2345.61	3639.78	3434.84	1678.66	2316.19	2804.26	2595.67	2041.43	4339.78	2419.85	2342.67	4069.92	5141.84	1994.98	1302.35	4599.57	1460.71	5351.88	2583.62
FLJ13125	0	52.74	59.49	57.56	65.4	34.05	52.06	26.07	38.16	27.32	33.19	38.57	54.33	85.14	58.69	56.33	33.71	56.8	17.26	45.98
APBB2	323	48.07	45.04	33.24	24.99	48.31	47.67	49.37	58.87	40.48	51.43	98.79	17.56	24.25	68.88	35	51.65	113.25	31.15	75.58
PRG1	5552	240.79	108.68	359.34	391.08	796.54	440.36	385.27	294.35	207.65	573.58	275.38	165.54	349.26	307.39	323.37	214.8	322.67	158.94	263.71
GBP1	2633	91.22	31.2	121.44	136.74	65.21	98.99	87.5	85.56	51	243.74	123.94	17.88	70.3	118.56	53.75	61.73	74.98	51.7	66.62
ALEX2	9823	118.17	239.73	202.06	14.47	97.27	264.08	328.76	142.98	174.7	161.15	236	193.08	300.63	70.22	60.25	121.19	178.16	372.69	173.39
CD53	963	319.47	206	513.29	503.92	519.43	598.53	492.39	467.93	177.01	784.45	360.55	224.26	497.2	421.37	433.11	165.92	288.14	325.24	294.12
VCAM1	7412	152.19	181.39	123.79	448.19	108.34	221.91	95.28	157.96	67.57	216.94	209.9	152.46	249.63	105.27	156	103.3	101.45	141	172.57
MAPT	4137	54.46	199.43	183.09	138.42	116.73	38.18	156.33	139	116.91	74.11	65.54	115.86	8.02	76.83	64.57	93.37	129.56	251.67	73.96
EGR2	1959	31.06	46.05	14.27	72.11	11.96	90.28	67.5	78.31	31.38	58.46	27.69	23.66	33.9	52.19	28.48	41.92	32.9	41.86	50.13
TDO2	6999	31.45	12.83	19.66	64.21	13.42	35.06	28.59	66.38	26.96	153.22	28.1	22.05	39.81	27.75	46.93	27.35	37.39	44.36	29.67
ADAMDEC1	27299	41.24	14.42	32.7	69.35	31.16	56.27	43.94	108.26	17.18	162.18	83.74	23.39	37.97	40.79	23.12	2.88	58.24	22.45	84.74
TFEC	22797	38.05	10.69	51.17	50.12	57.85	46.59	49.19	37.14	35.73	80.07	27.21	27.05	42.73	58.17	34.73	17.36	26.46	50.9	20.24
BTF3	689	2121.94	1920.59	1342.83	624.75	1513	1741.83	1545.7	1715.19	2119.89	990.84	1727.25	1971.02	1425.51	1716.47	678.94	2354.83	1557.4	2339.94	1394.59
FLNB	2317	275.11	352.27	97.71	428.84	133.56	143.52	325.36	174.02	148.04	202.09	290.04	210.99	180.4	196.97	141.08	429.07	184.34	301.53	174.26
TFRC	7037	237.29	850.47	662.9	1645.21	888.91	322.78	441.17	399.35	1216.07	825.41	376.29	528.61	795.16	272.05	707.83	518.43	562.71	769.01	443.94
EIF4B	1975	584.47	877.4	529.33	680.26	518.58	403.96	513.76	616.71	816.48	473.45	907.23	1054.74	510.02	397.34	400.48	1064.83	421.36	1412.24	492.9
MAPK3	5595	183.26	154.63	88.47	218.46	303.51	101.63	159.31	89.61	131.57	70.71	451.12	138.54	123.06	133.21	64.38	118.02	166.78	129.17	115.06
LOC161291	161291	145.61	264.44	154.03	107.84	216.37	105.47	280.67	166.07	135.86	90.09	261.9	293.83	105.28	193.91	87	141.06	210.92	187.98	44.08
SLC1A1	6505	25.26	582.23	279.86	20.26	61.58	84.05	39.42	57.6	98.53	57.73	30.67	175.95	51.14	321.76	39.73	125.5	311.26	283.12	34.19
MST4	51765	34.94	73	55.25	56.53	43.31	45.65	70.24	84.76	67.91	91.51	25.25	42.6	155.9	54.12	48.6	42.92	125.99	106.36	56.85
BLAME	56833	44.7	19.87	72.62	386.48	64.59	79.16	57.75	67.52	36.22	153.57	69.95	16.53	106.21	53.5	22.25	28.36	44.65	41.37	59.6
NME7	29922	58.09	122.15	108.49	12.81	58.79	79.24	132.33	92.5	67.87	44.72	55.88	105.41	79.3	64.78	53.55	53.71	176.16	127.76	61.71
FHL1	2273	128.07	53.78	23.58	11.43	102.26	64.8	125.84	55.31	34.98	17.47	28.4	60.66	34.58	69.23	28.49	1.3	19.29	28.19	82.11
FMOD	2331	403.83	385.69	155.1	190.39	331.95	364.18	348.86	272.8	152.3	306.6	251.3	110.71	266.97	327.99	142.67	221.83	174.65	502.25	398.58
CLDN5	7122	103.02	37.21	50.25	48.82	106.02	92.62	109.76	33.7	50.86	46.21	71.41	44.78	33.13	90.16	39.46	41.13	35.13	23.75	41.05

BC1132	Gene	Locus_Link	BC1096	BC1129	BC1130	BC1131	BC1256	BC1446	BC1116	BC1415	BC1141	BC1495	BC1497	BC1160	BC1038	BC1095	BC1024	BC1101	BC1139
140.29	CCNB2	9133	66.69	91.15	43.47	457.98	217.13	45.57	102.65	193.18	119.43	29.95	216.56	94.36	349.19	33.05	146.35	126.1	83.67
134.38	E2-EPF	27338	171.17	170.67	72.08	781.63	252.19	105.58	117.83	241.33	205.91	89.39	380.94	226.62	617.54	78.04	1154.79	397.19	356.79
55.88	MAD2L1	4085	86.57	26.01	39.01	197.27	58.38	67.1	48.94	89.27	41.96	27.34	209.22	70.55	177.46	21.86	51.52	221.38	36.26
211.56	DKC1	1736	356.48	209.28	391.81	610.79	476.31	283.08	320.01	312.57	336.61	233.2	457.54	462.28	431.35	318.9	537.54	408.34	248.08
61.87	PMSCL1	5393	56.49	22.96	45.58	108.7	119.07	34.3	34.09	91.3	39.57	20.84	128.78	83.9	87.22	19.17	34.19	140.96	25.86
446.26	KPNA2	3838	469.38	282.82	228.71	1149.3	351.46	225.64	308.07	359.99	446.81	187.34	466.27	589.67	712.32	248.15	326.05	355.96	412.24
91.99	CSE1L	1434	159.63	132.53	205.03	143.47	189.12	136.71	193.93	117.77	163.59	161.8	210.25	184.59	643.29	126.22	126.23	202.38	249.74
16.45	MGC3103	78999	121	107.19	11.86	57.5	134.63	56.53	31.34	25.99	84.83	50.76	134.24	65.33	69.52	10.05	103.7	69.52	25.25
145.58	PAI-RBP1	26135	262.63	634.74	525.35	372.36	658.8	417.83	340.19	630.74	309.37	497.27	307.41	124.34	482.65	331.65	264.43	366.41	306.37
204.72	RHEB2	6009	171.49	253.94	244.56	193.54	324.03	295.49	233.34	245.55	145.11	281.62	431.09	245.68	203.77	175.39	254.57	402.62	166.5
171.99	HDAC2	3066	93.78	390.44	271.22	90.14	480.04	143.95	149.94	307.47	164.08	200.68	139.17	181.02	123.45	150.58	63.77	188.74	280.72
159.29	PCMT1	5110	144.25	313.41	331.91	288.47	314.33	282.4	393.92	247.1	404.93	241.98	271.9	480.51	157.48	251.47	355.75	431.96	378.78
11.32	KIAA0056	23310	56.32	79.12	49.45	35.46	59.23	44.92	47.65	41.29	23.34	39.29	53.65	40.8	116.99	61.54	50.13	72.91	32.87
668.91	RAB31	11031	362.12	954.59	615.74	372.16	535.61	798.54	1315.07	752.02	777.5	649.36	483.4	59.03	313.14	538.45	139.44	345.69	374.12
353.04	FLJ20273	54502	603.4	199.36	621.71	360.74	162.34	306.65	773.23	155.5	120.87	467.64	495.82	402.91	379.5	526.15	72.79	605.23	371.46
142.27	GRP	2922	57.84	102.71	9.3	81.19	72.05	145.66	121.82	276.6	97.07	82.5	153.24	8.96	5.21	151.14	52.45	86.53	65.45
145.13	AD-017	55830	212.14	207.66	279.69	242.18	237.35	274.42	284.61	229.41	97.4	262.32	205.43	251.73	171.53	317.29	157.95	249.1	155.81
257.08	IMPDH2	3615	420.96	397.33	981.65	679.39	566.37	654.42	1405.82	530.42	837.01	339.95	739.39	1025.01	841.92	858.34	1628.67	944.95	559.98
304.05	FHL2	2274	229.59	332.95	474.77	253.37	345.22	321.61	836.02	356.54	381.7	354.01	157.2	301.49	56.91	319.52	65.41	169.16	112.31
237.83	FLJ22642	0	151.6	224.17	173.23	199.37	116.28	187.68	172.27	168.4	167.23	196.63	219.1	138.74	189.72	137.83	201.77	165.17	156.89
99.31	DCTN4	51164	117.19	75.56	104.87	70.49	63.62	108.97	95.17	81.45	63.72	133.1	159.53	45.41	106.81	113.09	71.27	140.01	107.96
51.63	DDB2	1643	69.55	16.69	88.23	74.38	46.92	49.89	114.18	82.25	69.47	89.29	85.77	52.4	53.26	68.37	76.25	58.41	25.56
490.31	YR-29	10412	399	496.45	898.16	591.75	292.59	844.53	1062.02	497.17	762.83	589.76	888.59	324.32	646.27	508.11	321.74	677.29	838.52
542.87	SSR1	6745	193.97	688.77	663.27	450.57	877.56	614.75	511.38	730.16	799.33	843.47	439.28	1045.46	721.3	440.22	222.96	369.49	860.84
1831.34	IGFBP4	3487	750.12	1204.38	1961.12	688.06	893.26	1661.09	1602.7	913.73	961.75	1345.28	1515.52	838.53	801.03	972.03	467.96	630.02	757.99
198.32	SMC1L1	8243	363.15	206.06	241	355.5	335.53	189.21	186.58	208.37	196.85	257.5	416.51	217.13	466.79	173.01	213.17	289.54	317.72
224.93	ARL3	403	214.05	144.05	245.76	163.73	160.93	207.14	282.9	144.4	156.34	125.79	211.92	185.95	222.6	175.83	219.38	173.34	433.98
58.31	SEMA3C	10512	53.99	59.73	30.77	78.1	25.82	35.41	68.73	34.24	46.68	48.48	281.36	38.21	52.46	87.79	20.57	57.4	37.84
55.32	XPA	7507	71.84	62.71	101.67	99.61	51.63	75.02	80.74	49.25	83.54	113.97	59.89	65.05	47.36	61.43	49.25	78.57	87.15
57.11	PLA2G7	7941	37.83	79.19	16.36	110.08	57.66	168.38	12.81	55.15	85.1	15.13	58.79	31.26	147.22	30.41	24.67	36.57	15.91
18.18	BTBD2	55643	67.46	82.3	117.8	72.53	108.31	64.7	130.39	95.01	55.11	77.81	96.98	121.99	76.13	81.58	60.43	60.61	164.21
186.48	LIG1	26018	241.66	202.34	560.5	629.02	142.41	193.82	334.08	111.3	235.5	203.06	174.8	374.25	129.11	412.52	137.16	174.93	231.12
60.54	TNRC15	26058	126.84	80.23	85.89	54.62	57.28	70.69	110.38	62.77	72.41	80.02	81.27	93.1	92.78	142.32	59.9	111.76	97.73
2003.72	RPL17	6139	1227.26	2093	4766.87	3043.14	2421.88	3101.02	4491.22	2622.06	3205.71	1311.37	2571.08	3115.5	1996.86	2999	2500.19	1878.32	2291.77
26.71	FLJ13125	0	71.53	80.59	37.52	79.7	53.72	44.47	54.6	35.47	62.3	56.31	77.85	63.51	46.98	34.45	19.41	47	19.06
65.91	APBB2	323	66.09	60.49	60.07	143.44	20.45	81.17	57.72	61.16	26.24	68.92	100.87	26.11	76.83	41.1	13.33	29.32	75.11
341.47	PRG1	5552	347.35	445.48	291.51	242.18	359.91	364.64	211.85	473.72	419.48	532.19	373.19	106.5	509.52	142.12	182.24	332.8	319.97
71.35	GBP1	2633	59.71	105.6	45.41	79.8	84.56	69.29	40.45	226.42	155.77	161.49	52.94	32.71	104.82	51.27	41	84.19	97.4
88.41	ALEX2	9823	170.26	113.76	186.02	187.3	112.34	377.67	266.76	138.02	64.77	259.28	94.22	280.79	93.06	280.4	10.27	205.89	219.22
410	CD53	963	461.46	815.04	237.14	296.67	301.37	474.97	188.82	504.55	622.79	394.75	407.44	182.19	575.26	202.23	326.28	454.03	316.06
98.74	VCAM1	7412	125.89	137.37	125.56	126.42	112.48	176.32	96.35	439.24	160.05	194.03	139.41	88.5	170.89	201.96	132.79	113.22	60.94
6.85	MAPT	4137	250.36	39.82	94.34	92.15	66.26	129.45	114.01	25.67	50.85	127.18	79.39	163.46	15.38	112.52	187.41	104.34	102.92
63.43	EGR2	1959	47.93	42.71	77.66	31.7	60.98	101.45	55.89	80.25	27.87	32.28	60.68	59.95	80.76	23.35	12.74	21.83	21.28
54.55	TDO2	6999	52.37	46.45	20.12	39.68	29.26	28.99	22.64	28.34	53.91	14.52	9.62	10.73	22.71	18.25	20.47	39.91	25.27
60.91	ADAMDEC1	27299	84.72	101.23	20.81	58.39	91.16	47.95	30.11	77.83	119.79	30.49	108.95	17.93	267.43	18.68	56.3	68.23	35.08
39.31	TFEC	22797	47.67	34.03	19.2	38.69	20.3	54.88	23.38	51.53	61.27	32.8	33.63	14.49	50.86	27.05	15.34	52.8	36.32
1432.31	BTF3	689	1777.35	988.82	2558.43	1807.58	790.68	2063.23	2020.2	1125.36	1396.14	1301.55	1760.61	1158.15	2052.32	1260.76	1989.83	1694.21	2311.54
129.34	FLNB	2317	671.41	129.17	303.46	262.64	536.74	134.83	485.13	333.6	176.61	131.54	573.46	372.01	212.5	276.17	260.34	208.94	443.44
713.89	TFRC	7037	174.61	950.12	373.46	1309.93	833.82	465.69	865.32	632.3	518.7	575.82	286.58	413.4	1690.96	285.99	536.96	753.62	521.18
416.84	EIF4B	1975	476.93	459.75	701.56	524.86	427.94	514.19	597.85	416.98	1038.93	511.05	800.29	877.6	732.04	743.9	415.01	671.29	495.75
96.36	MAPK3	5595	188.89	96.8	153.35	111.49	85.93	117.77	136.44	82.54	76.32	239.15	129.71	171.66	194.19	113.96	214.14	173.11	118.61
124.84	LOC161291	161291	199.9	75.64	202.76	92.2	118.7	143.22	198.47	96.84	137.31	253.79	114.79	162.73	88.29	403.02	54.02	138.56	229.22
100.47	SLC1A1	6505	46.41	145.33	284.51	235.47	100.92	262.06	38.09	32.58	212.4	549.53	33.77	282.24	177.48	442.11	3.77	19.85	676.39
54.1	MST4	51765	68.29	60.1	59.25	251.3	118.9	51.35	58.3	54.8	121.24	73.11	94.5	106.51	215.14	46.64	35.29	95.17	79.81
66.26	BLAME	56833	49.97	78.17	19.87	51.07	60.74	33.23	19.23	122.36	161.84	79.55	39.03	15.67	111.37	20.31	79.09	63.01	15.95
65.37	NME7	29922	93.71	64.19	90.44	69.28	87.67	99.5	74.02	53.53	86.65	107.23	63.81	72.26	47.52	165.67	34.92	108.53	102.04
70.59	FHL1	2273	23.73	99.38	68.43	33.98	49.94	121.85	47.18	94.55	4.16	59.13	58.05	29.55	63.36	88.21	10.29	19.63	17.74
280.56	FMOD	2331	123.63	367.17	465.67	132.13	211.88	421.03	290.48	467.58	247.43	199.83	249.8	249.19	86.33	281.12	496.6	136.23	138.12
65	CLDN5	7122	14.18	75.49	53.49	48.31	23.62	88.89	54.57	57.05	9.24	69.09	14.36	35.34	43.41	56.87	49.35	39.88	34.71
