# Supplementary material for: Predictors of primary breast cancers responsiveness to preoperative Epirubicin/Cyclophosphamide-based chemotherapy: translation of microarray data into clinically useful predictive signatures
Source: J Transl Med. 2005 Aug 9;3:32. doi: 10.1186/1479-5876-3-32 (PMC1201176; doi:10.1186/1479-5876-3-32)
Supplement: Additional File 10 — contains raw data for 59 genes and 83 tumors in training and test cohorts, respectively. [file 1479-5876-3-32-S10.pdf]

Gene	Locus_Link	BC1843	BC1850	BC1862w	BC1871	BC1869	BC1864	BC1421	BC1870	BC1861	BC1879	BC1866	BC1837	BC1838	BC1842	BC1834w	BC1858w	BC1880	BC1881	BC1849
CCNB2	9133	80.49	42.57	99.42	55.22	262.21	220.2	328.21	276.59	198.04	214.59	55.78	180.53	113.07	111.53	93.94	69.38	131.13	142.11	52.59
E2-EPF	27338	185.75	121.36	156.7	202.89	1363.55	756.79	353.27	825.18	166.68	819.37	123.59	420.97	1283.58	278.5	118.82	90.94	211.56	102.16	93.11
MAD2L1	4085	34.52	23.64	47.86	48.56	158.04	115.78	87.81	116.79	102.5	110.32	19.03	80.26	65.01	34.21	59.44	27.59	36.32	50.62	32.99
DKC1	1736	276.92	273.23	287.28	359.52	508.77	412.24	211.25	707.47	362.55	554.07	274.66	559.72	300.1	270.01	244.04	332.21	358.57	314.8	255.56
PMSCL1	5393	43.71	19.41	22.45	26.97	74.96	101.99	67.96	89.67	47.73	37.54	32.11	42.86	40.29	32.15	42.46	21.01	34.42	38.98	21.94
KPNA2	3838	247.98	89.37	171.31	236.34	555.52	592.72	480.17	573.08	381.38	812.16	226.19	201.2	332.5	136.5	308.6	126.93	234.75	316.19	200.13
CSE1L	1434	110.39	109.45	115.38	124.49	128.09	123.57	219.69	448.39	109.89	255.69	141.87	123.2	218.9	185.41	204.34	126.12	156.74	87.12	76.69
MGC3103	78999	59.49	59.58	26.36	47.13	117.31	61.03	29.51	114.99	37.37	61.87	25.29	69.54	57.18	42.25	55.58	14.65	68.54	10.58	31.35
PAI-RBP1	26135	194.61	327.15	282.57	539.75	592.84	432.37	521.49	830.17	344.08	540.06	563.78	263.75	234.5	240.8	249.62	324.39	448.07	550.46	154.52
RHEB2	6009	88.89	178.64	109.54	178.57	301.31	200.76	307.1	350.27	118.23	207.05	160.22	112.82	124.13	128.89	211.23	119.54	196.04	193.92	62.8
HDAC2	3066	116.12	100.06	79.61	172.09	431.15	230.14	489.91	233.84	177.82	124.28	108.78	130.44	60.3	112.35	203.23	66.17	164.52	106.54	70.39
PCMT1	5110	140.1	291.48	186.52	205.2	274.43	154.66	222.57	212.19	197.4	163.79	217.7	150.29	135.23	159.77	200.05	139.23	219.31	130.09	103.14
KIAA0056	23310	30.67	11.45	35.92	28.04	63.17	59.21	83.98	56.61	26.8	56.85	31.97	59.47	32.27	78.94	51.08	48.61	47.06	60.28	135.87
RAB31	11031	727.51	943.96	523.89	684.79	194.16	234.96	206.18	301.09	286.53	204.72	529.65	220.21	232.8	645.57	295.97	377.01	466.71	512.4	199.03
FLJ20273	54502	550.49	221.26	242.46	374.89	115.21	158.08	360.17	257.47	183.31	227.3	362.6	187.7	870.06	248.82	262.41	587.47	324.51	605.01	784.36
GRP	2922	82.62	254.08	139.42	124.87	3.39	4.15	111.87	10.05	31.41	5.22	54.4	10.28	10.05	91.44	94.94	99.19	115.75	85.62	17.24
AD-017	55830	253.97	303.14	183.98	268.25	140.08	117.17	198.42	73.2	125.29	272.72	184.53	211.08	217.7	203.65	106.09	251.51	173.69	226.34	210.57
IMPDH2	3615	1102.25	508.61	539.02	1069.05	654.54	401.35	735.13	158.15	341.62	1480.8	450.44	506.79	877.74	493.93	280.81	667.71	503.58	593.23	1059.51
FHL2	2274	257.76	245.88	287.7	437.68	136.5	134.45	107.38	198.05	297.14	45.17	116.2	97.78	117.93	271.47	212.41	387.29	378.24	143.39	115.6
FLJ22642		220.66	239.92	166.6	141.4	194.53	124.35	339.19	98.42	108.55	156.04	105.96	135.36	109.75	96	204.95	164.99	253.32	192.12	103.04
DCTN4	51164	112.77	137.55	146.66	109.96	93.82	91.09	113.09	115.64	87.63	151.86	104.44	70.71	147.26	121.88	89.36	102	147.21	81.57	148.29
DDB2	1643	49.28	59.89	122.4	69.39	24.02	51.7	68.39	35.35	50.06	78.92	46.08	45.04	50.35	51.62	40.37	69.98	54.75	59.06	65.63
YR-29	10412	575.51	790.42	431.65	925.63	396.15	471.13	436.39	401.76	663.91	827.86	591.95	624.42	671.69	482.8	785.63	572.58	840.67	786.15	538.83
SSR1	6745	426.19	530.12	427.21	747.96	854.25	380.07	997.9	1033.53	1272.68	606.19	609.09	477.58	414.69	662.47	475.84	637.37	506.07	782.02	282.08
IGFBP4	3487	1643.53	3045.34	1941.98	2179.71	508.88	682.45	511.95	768.39	2250.05	1360.49	951.03	1270.12	286.29	1137.32	1189.38	1697.8	1591.64	1223.29	1689.41
SMC1L1	8243	311.26	172.88	140.22	215.24	218.5	259.21	454.41	335.82	341.08	282.02	229.22	148.13	138.04	232.06	178.93	168.2	273.08	222.77	88.18
ARL3	403	272.29	208.11	158.77	198.35	232.4	126.65	78	168.05	127.52	182.83	176	97.92	323.61	149.31	146.28	243.13	246.66	94.95	196.13
SEMA3C	10512	92.26	92.75	114.3	55.79	33.29	34.43	43.34	14.31	33.84	90.88	59.4	25.91	98.96	74.62	79.6	71.35	129.09	35.55	64.5
XPA	7507	72.71	61	77.08	100.34	40.13	56.5	69.51	52.32	54.76	102.98	87.55	80.98	82.27	51.59	59.39	102.9	110.07	63.52	134.19
PLA2G7	7941	62.88	14.03	77.53	21.55	134.45	148.34	47.33	123.19	66.18	31.78	128.93	158.44	17.47	26.45	52.79	12.21	28.1	187.58	61.4
BTBD2	55643	97.7	151.04	77.29	75.04	114.89	49.9	54.47	57.18	108.15	86.6	57.26	175.23	290.02	148.19	28.24	104.04	72.58	105.39	145.1
LIG1	26018	80.33	150.8	189.53	332.71	78.88	105.36	261.57	181.2	118.91	742.57	250.07	102.22	159.08	124.64	219.93	455.86	101.49	268.13	251.47
TNRC15	26058	99.46	127.53	139.83	98.86	132.36	145.66	75.84	106.01	101.71	128.03	77.23	92.4	170.4	263.8	76.12	108.47	130.17	91.65	107.57
RPL17	6139	2242.18	2536.62	1847.41	3377	1992.75	2529.37	1346.23	2524.89	1933.55	1315.78	2442.95	3480.69	1936.64	3258.34	2444.7	2367.1	3560.91	2117.88	2503.03
FLJ13125		30.95	28.93	5.45	25.19	89.07	30.78	16.8	91.2	30.59	28.16	24.36	27.96	41.02	26.22	26.8	19.92	34.74	25.14	24.25
APBB2	323	133.32	79.69	87.14	54.69	15.78	29.02	38.87	21.9	40.97	93.15	38.27	22.01	39.14	61.67	26.05	73.82	61.15	47.55	74.69
PRG1	5552	270.94	218.97	178.63	281.92	346.64	421.45	416.32	349.06	365.04	131.6	614.75	621.2	72.34	292.44	404.69	153.27	297.47	309.64	362.25
GBP1	2633	45.25	60.7	57.84	57.7	72.53	130.44	377.7	213.69	109.96	29.13	203.64	146.05	11.12	115.58	158.27	24.86	52.12	28.63	24.1
ALEX2	9823	173.38	274.44	213.3	230.87	204.49	151.98	60.27	193.66	240.46	302.66	138.09	74.59	324.86	171.08	309.27	364.18	227.56	187.76	115.49
CD53	963	442.42	233.51	217.4	334.4	407.32	745.46	473.19	421.2	658.48	169.84	1130.7	1273.61	159.34	455.65	542.56	216.37	437.02	600.66	359.77
VCAM1	7412	261.32	47.81	181.93	235.42	123.28	156.55	248.77	211.25	266.73	88.35	438.25	349.8	20.98	346.45	204.22	91.06	145.01	122.35	184.34
MAPT	4137	61.82	106.12	72.27	136.59	13.86	20.63	41.82	5.39	17.83	113.72	79.29	26.94	399.46	21.29	11.13	318.88	97.26	94.06	6.89
EGR2	1959	49.55	42.29	43.11	234.6	21.7	43.37	40.2	44.41	52.02	13.77	82.65	50.84	6.55	23.2	64.75	32.53	41.17	31.88	25.71
TDO2	6999	22.48	17.97	40.62	25.92	76.61	61.88	56.34	50.55	34.46	21.81	77.44	58.88	14.61	15	34.68	20.27	35.97	238.05	37.08
ADAMDEC1	27299	23.48	26.25	26	53.94	306.05	194.57	176.8	238.04	71.78	51.79	493.4	516.56	7.91	131.07	231.61	9.34	48.98	98.86	65.14
TFEC	22797	36.01	17.7	46.95	27.06	38.72	86.71	63.6	69.52	41.69	22.86	134.72	122.26	18.89	45.54	71.29	13.48	28.54	49.66	46.58
BTF3	689	1756.73	2183.63	1140.38	1897.57	891.42	1114.74	1232.51	621.63	966.41	2010.84	1920.45	1650.11	1957.2	1385.98	1654.7	1771.28	1732.41	1467.28	1872.32
FLNB	2317	309.54	206.13	105.91	158.94	173.77	148.45	355.38	196.15	146.18	783.61	151.15	170.41	464.62	181.5	87.89	348.76	64.07	194.23	467.4
TFRC	7037	348.12	129.5	240.72	425.45	1299.71	1113.65	678.13	1094.31	1013.54	134.85	700.86	593.51	423.41	1510.49	543.59	692.38	298.97	959.82	164.43
EIF4B	1975	520.78	677.53	617.5	691.52	439.75	337.84	355.13	634.71	533.5	480.34	494.12	644.09	661.13	624.33	364.96	790.39	610.07	406.84	543.24
MAPK3	5595	131.27	150.47	97.75	116.24	69.69	43.58	79.85	84.94	85.31	214.93	91.61	66.1	183.93	69.5	84.44	150.83	110.12	86.08	207.66
LOC161291	161291	150.3	194.57	179.77	161.68	59.21	45.25	167.25	78.94	89.85	196.18	206.56	86.17	355.45	90.74	176.43	219.44	104.35	205.87	129.11
SLC1A1	6505	22.36	37.7	403.3	143.5	18.29	34.1	84.47	20.26	48.83	19.1	593.4	15.7	12.17	76.35	167.94	76.26	116.1	91.01	16.8
MST4	51765	25.97	13.8	31.21	43.1	147.52	30.54	65.14	85.67	69.16	72.69	40.34	85.12	29.72	51.52	51.25	17.52	58.83	34.07	35.92
BLAME	56833	82.36	30.35	24.04	40.71	118.29	107.57	70	131.78	120.28	17.96	201.52	229.25	2.16	123.54	132.94	20.05	48.96	85.47	85.85
NME7	29922	72.03	66.32	173.08	130.51	95.26	87.3	93.16	112.18	56.44	74.69	85.18	54.09	88.31	119.12	172.5	137.46	64.16	61.54	91.79
FHL1	2273	71.26	89.19	104.37	68.58	27.36	53.89	19.86	30.53	147.27	26.25	29.97	55.07	12.9	30.29	25.02	48.5	79.36	47.64	65.54
FMOD	2331	160.88	408.69	610.59	489.56	132.07	125.63	205.94	75.12	604.51	83.16	238.57	198.28	47.73	275.14	231.2	569	549.35	252.06	217.56
CLDN5	7122	56.61	42.69	41.52	65.11	38.08	48.51	10.72	28.1	71.06	27.95	57.83	132.94	21.52	22.03	45.78	72	95.3	45.41	74.37

Gene	Locus_Link	BC1839	BC1513	BC1877	BC1853w	BC1448	BC1134	BC1840	BC1848
CCNB2	9133	139.64	44.66	46.29	56.34	106.2	100.75	178.99	140.1
E2-EPF	27338	201.55	92.06	194.34	110.29	120.62	330.03	796.69	396.14
MAD2L1	4085	34.19	39.59	39.85	24.26	45.86	63.1	90.29	47.11
DKC1	1736	829.79	227.27	286.24	270.73	300.04	337.12	299.3	215.07
PMSCL1	5393	38.34	30.4	32.8	16.47	41.46	50.91	57.02	30.39
KPNA2	3838	121.84	213.67	194.01	191.47	224.48	193.04	350.4	605.47
CSE1L	1434	88.8	175.45	136.49	132.78	101.28	131.97	182.18	63.99
MGC3103	78999	4.98	39.38	71.73	39.72	6.15	63.39	61.68	45.01
PAI-RBP1	26135	142.3	436.42	601.56	257.78	578.75	284.7	151.97	284.44
RHEB2	6009	112.17	220.26	224.07	160.79	111.9	205.07	172.81	111.75
HDAC2	3066	97.62	95.71	329.67	79.6	51.22	117.33	94.21	118.61
PCMT1	5110	79.37	130.25	212.64	175.13	105.16	204.73	158.35	112.76
KIAA0056	23310	96.55	11.3	22.75	39.59	84.61	62.26	63.7	52.05
RAB31	11031	295.95	275.37	412.29	430.52	307.76	568.52	566.06	79.58
FLJ20273	54502	577.79	684.7	473.63	566.7	159.9	624.34	711.95	371.7
GRP	2922	57.69	51.11	130.24	139.46	7.01	54.93	13.83	6.03
AD-017	55830	242.34	290.7	217.07	361.84	258.2	176.42	117.34	130.24
IMPDH2	3615	1328.27	705.56	782.24	1140.92	636.49	613.15	900.12	316.95
FHL2	2274	169.1	390.1	340.45	196.7	147.72	144.14	116.45	145.95
FLJ22642		282.77	243.82	163.99	195.88	80.6	192.61	97.7	86.97
DCTN4	51164	105.16	91.63	183.56	140.56	97.23	133.36	102.25	116.73
DDB2	1643	41.93	85.8	41.51	50.11	91.65	143.96	45.7	55.34
YR-29	10412	688.74	974.64	817.47	568.93	685.34	581.8	532.89	491.06
SSR1	6745	370.06	755	394.79	364.57	705.83	548.43	271.89	434.23
IGFBP4	3487	804.62	1725.9	1415.43	1788.16	3126.15	1593.29	2175.05	715.45
SMC1L1	8243	109.81	178.54	191.89	143.87	153.45	247.69	229.86	159.71
ARL3	403	173.34	207.51	252.17	176.91	110.72	347.18	345.36	130.96
SEMA3C	10512	20.88	57.73	79.59	114.71	124.2	49.7	66.25	63.05
XPA	7507	78.17	121.15	79.55	131.81	103.46	84.27	63.75	67.52
PLA2G7	7941	15.08	35.59	10.03	8.11	152.95	31.34	31.99	99.98
BTBD2	55643	150.45	188.03	120.63	190.32	95.96	132.9	239.55	74.1
LIG1	26018	269.36	410.82	334.16	205.24	156.89	415.69	193.34	84.99
TNRC15	26058	110.26	78.46	133.69	145.65	102.92	131.95	113.29	100.2
RPL17	6139	2104.2	2159.08	3288.88	1849.77	3040.65	1686.94	2547.82	1822.11
FLJ13125		22.65	67.27	47.25	37.92	14.39	36.09	51.84	16.96
APBB2	323	53.17	87.68	58.42	73.06	38.91	42.89	112.24	44.94
PRG1	5552	132.63	360.72	189.3	229.03	459.83	224.42	74.17	501.28
GBP1	2633	37.47	164.08	24.5	28.13	109.63	53.91	13.17	145.14
ALEX2	9823	54.18	399.95	361.12	292.19	139.7	115.42	68.51	254.67
CD53	963	199	390.56	237.09	188.38	836.07	305.18	124.58	826.5
VCAM1	7412	117.63	211.75	42.81	34.44	178.65	90.11	97.64	246.56
MAPT	4137	66.33	147.43	143.96	136.61	29.9	189.92	597.12	17.8
EGR2	1959	29.27	29.75	24.13	37.71	73.66	21.84	16.22	52.56
TDO2	6999	24.3	10.91	10.65	13.72	47.27	19.96	16.55	43.38
ADAMDEC1	27299	13.11	21.49	11.69	23.17	214.18	31.82	10.6	219.64
TFEC	22797	22.2	18.61	17.31	17.48	65.87	26.21	18.09	76.66
BTF3	689	1703.12	1765.61	1717.88	1432.26	1492.68	1597.12	1804.59	1574.69
FLNB	2317	837.66	292.54	313.49	316.52	55.69	632.33	932.01	130.03
TFRC	7037	377	256.88	335.9	187.51	538.38	599.37	248.07	632.45
EIF4B	1975	461.72	874.44	708.55	767.68	458.35	929.58	489	531.94
MAPK3	5595	127.36	194.34	147.14	251.39	102.07	505.99	201.3	127.44
LOC161291	161291	333.57	180.64	338.25	220.86	77.78	282.03	121.06	165.84
SLC1A1	6505	394.98	33.85	36.26	96.49	50.41	19.87	59.38	763.53
MST4	51765	16.16	42.72	36.77	13.24	53.12	34.78	39.41	51.03
BLAME	56833	38.73	37.13	17.4	33.07	110.72	56.85	5.74	172.32
NME7	29922	45.2	60.13	108.2	155.47	90.86	92.79	98.42	59.02
FHL1	2273	25.89	147.48	47.22	77.76	258.81	17.41	21.03	52.13
FMOD	2331	153.57	484.03	606.9	452.53	667.25	100.96	77.52	141.34
CLDN5	7122	28.94	108.47	55.21	19	141.65	54.15	34.62	61.64
